# Supplementary material for: Genetically Encoded Ratiometric pH Sensors for the Measurement of Intra- and Extracellular pH and Internalization Rates
Source: Biosensors (Basel). 2022 Apr 25;12(5):271. doi: 10.3390/bios12050271 (PMC9138566; doi:10.3390/bios12050271)
Supplement: Supplementary file 1 [file biosensors-12-00271-s001.zip › biosensors-1660845-supplementary.pdf]

biosensors

Supporting Information

Genetically Encoded Ratiometric pH Sensors for the Measurement of Intra- and Extracellular pH and Internalization Rates

Lennard Karsten [1], Lukas Goett-Zink [2], Julian Schmitz [3,4], Raimund Hoffrogge [5], Alexander Grünberger [3,4], Tilman Kottke [2] and Kristian M. Müller [1,*]

1 Cellular and Molecular Biotechnology, Faculty of Technology, Bielefeld University, 33615 Bielefeld, Germany; lennard.karsten@uni-bielefeld.de
2 Biophysical Chemistry and Diagnostics, Medical School OWL and Faculty of Chemistry, Bielefeld University, 33615 Bielefeld, Germany; lukas.goett-zink@uni-bielefeld.de (L.G.-Z.); tilman.kottke@uni-bielefeld.de (T.K.)
3 Multiscale Bioengineering, Faculty of Technology, Bielefeld University, 33615 Bielefeld, Germany; j.schmitz@uni-bielefeld.de (J.S.); alexander.gruenberger@uni-bielefeld.de (A.G.)
4 Center for Biotechnology (CeBiTec), Bielefeld University, 33615 Bielefeld, Germany
5 Cell Culture Technology, Faculty of Technology, Bielefeld University, 33615 Bielefeld, Germany; raimund.hoffrogge@uni-bielefeld.de
* Correspondence: kristian@syntbio.net

Citation: Karsten, L.; Goett-Zink, L.; Schmitz, J.; Hoffrogge, R.; Grünberger, A.; Kottke, T.; Müller, K.M. Genetically Encoded Ratiometric pH Sensors for the Measurement of Intra- and Extracellular pH and Internalization Rates. *Biosensors* **2022**, *12*, 271. https://doi.org/10.3390/bios12050271

Received: 15 March 2022
Accepted: 11 April 2022
Published: 25 April 2022

Publisher's Note: MDPI stays neutral with regard to jurisdictional claims in published maps and institutional affiliations.

Copyright: © 2022 by the authors. Licensee MDPI, Basel, Switzerland. This article is an open access article distributed under the terms and conditions of the Creative Commons Attribution (CC BY) license (https://creativecommons.org/licenses/by/4.0/).

Table of Content

1. Supplementary Figures and Tables 3

Figure S1. Expression of CoGFP_V0 in the cytosol of E. coli. 3

Figure S2. Expression of EGF-CoGFP_V0 in the cytosol of E. coli. 4

Figure S3. UV-Vis Spectrum of EGF-mCherry purified from E. coli after cytosolic expression. 5

Figure S4. pH-dependent fluorescence of CoGFP_V0 and EGF-CoGFP_V0 measured by a fluorescence plate reader. 5

Figure S5. pH-dependent 3D fluorescence spectra of CoGFP_V0 and EGF-CoGFP_V0. 6

Figure S6. Live-cell imaging of A431 cells incubated with EGF-CoGFP_V0. 7

Figure S7. Expression of EGF-CoGFP-mTagBFP2 in the cytosol of E. coli. 8

Table S1. List of identified proteins from MS/MS data evaluation. 8

Table S2. List of identified peptide groups. 9

Figure S8. Protein sequence of EGF-CoGFP-mTagBFP2. 13

Figure S9. Multiple protein sequence alignment of fluorescent proteins. 14

Figure S10. Expression of EGF-CoGFP-mCRISPRed in the cytosol of E. coli. 15

Figure S11. Binding specificity of EGF-CoGFP_V0 and EGF-CoGFP-mCRISPRed towards EGFR. 15

Figure S12. Expression of mTagBFP2 in the cytosol of E. coli. 16

Figure S13. Sequence alignment of different FRET linkers for tandem fluorescent proteins. 16

Figure S14. pH-dependent 3D fluorescence spectra of mTagBFP2. 17

Figure S15. Indirect determination of FRET efficiciency from 3D fluorescence spectra. 18

Table S3. Maturation times of fluorescent proteins from Entacmaea quadricolor. 18

Figure S16. Maturation assay of EGF-CoGFP-mCRISPRed. 20

Figure S17. Data acquisition for calibration curves of EGF-CoGFP-mTagBFP2 and EGF-CoGFP-mCRISPRed. 21

Table S4. Calculated variables for calibration curves of EGF-CoGFP-mTagBFP2 and EGF-CoGFP-mCRISPRed. 22

Figure S18. Live-cell imaging of untreated cells for autofluorescence correction. 22

Figure S19. Intracellular pH-mapping using FRET properties of EGF-CoGFP-mTagBFP2. 23

Figure S20. Luminal pH-gradients of endosomal and lysosomal vesicles. 24

Figure S21. Determination of transfection efficiency using flow cytometry. 25

2. Amino Acid Sequences 25

2.1. CoGFP_V0-His6 25

2.2. EGF-CoGFP-His6 25

2.3. EGF-CoGFP-mTagBFP2 26

2.4. EGF-CoGFP-mCRISPRed 26

References 26

1. Supplementary Figures and Tables

Figure S1. Expression of CoGFP_V0 in the cytosol of E. coli.

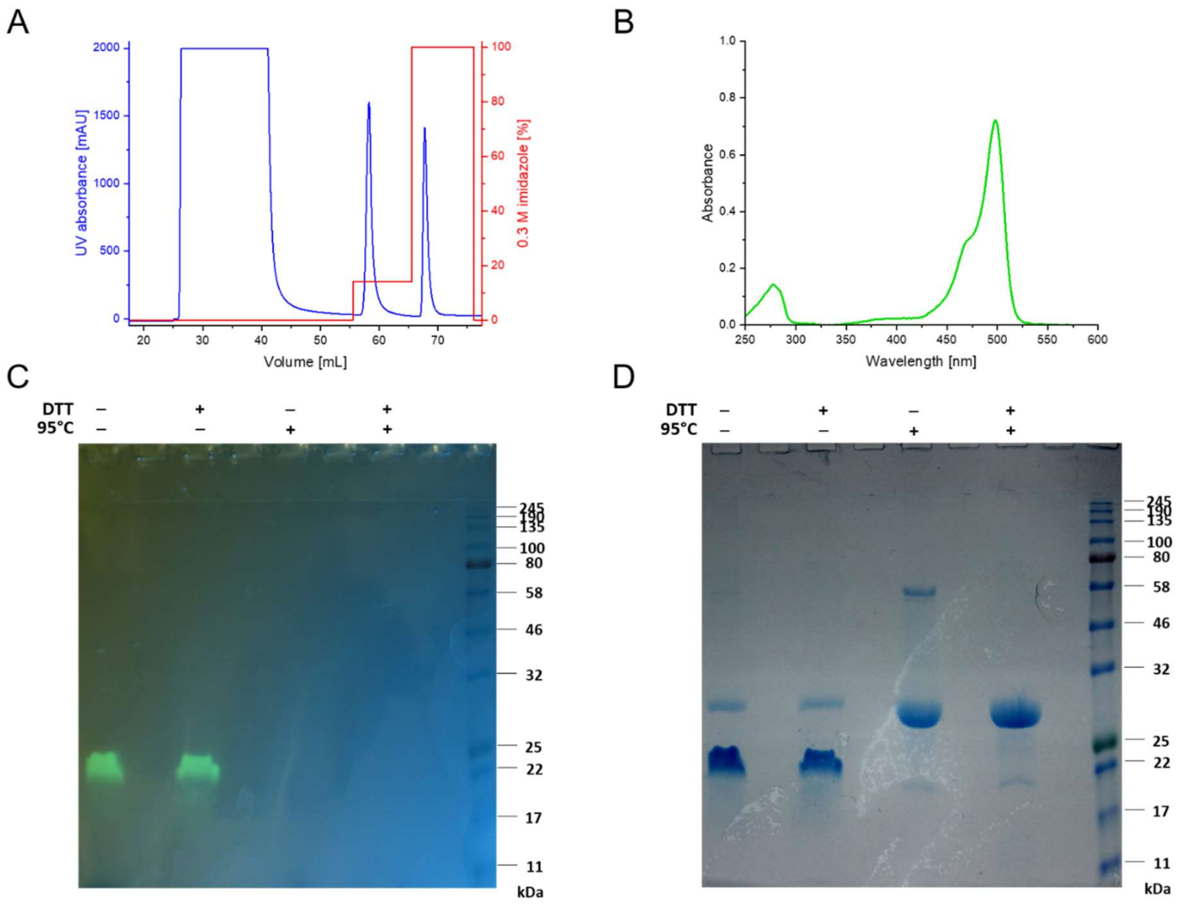

Figure S1. Expression of CoGFP_V0 in the cytosol of E. coli. (**A**) UV absorbance (blue) and conductivity (red) of IMAC (Ni-NTA) for purification of CoGFP_V0, which was expressed in the cytosol of *E. coli* BL21(DE3). (**B**) UV-Vis spectrum of purified CoGFP_V0 at pH 8. SDS PAGE of CoGFP_V0 under various conditions. (**C**-**D**) CoGFP_V0 was incubated with reducing (+DTT) or without reducing agent (–DTT) either at room temperature or at 95°C for 10 min before application to an SDS-PAGE. Images show the gel under white light exposure (**C**) before and (**D**) after Coomassie staining. The unstained gel visualizes the prestained marker and the functional CoGFP_V0 chromophore.

Figure S2. Expression of EGF-CoGFP_V0 in the cytosol of E. coli.

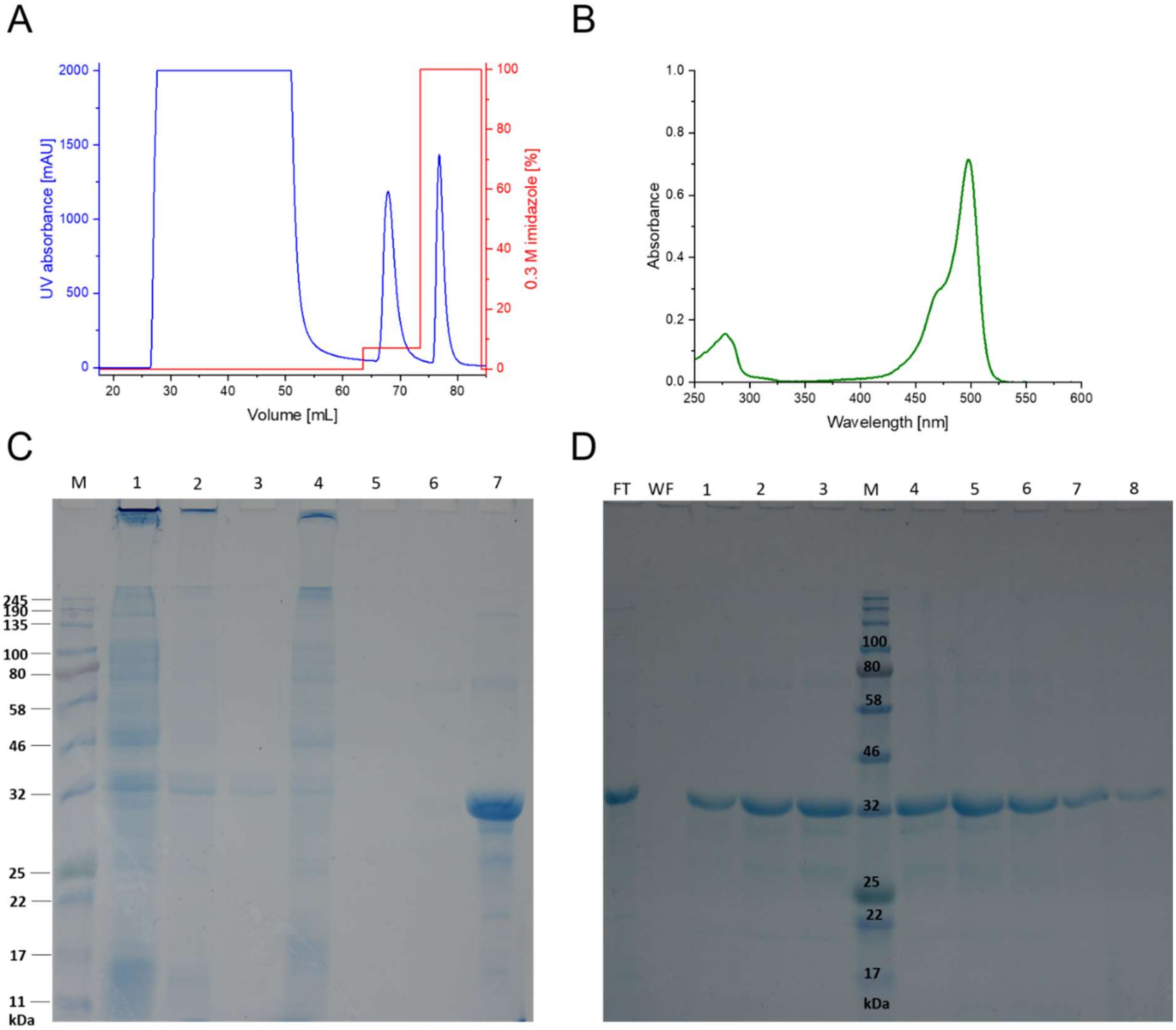

Figure S2. Expression of EGF-CoGFP_V0 in the cytosol of *E. coli*. (**A**) UV absorbance (blue) and conductivity (red) of IMAC (Ni-NTA) for purification of EGF-CoGFP_V0, which was expressed in the cytosol of *E. coli* BL21(DE3). (**B**) UV-Vis spectrum of purified EGF-CoGFP_V0 at pH 7.4. (**C**) Coomassie-stained reducing SDS gel after IMAC purification of EGF-CoGFP_V0. (1) Cell lysate before IPTG induction, (2) cell lysate after IPTG induction, (3) insoluble fraction, (4) column flow through, (5-6) wash fractions, (7) elution fraction, (M) molecular marker. (**D**) Ion exchange chromatography (IEX) was performed using an ÄKTA start protein purification system. The EGF-CoGFP_V0 (0.5 mL, 1.1 mg/mL) was applied to a self-packed Q Sepharose Fast Flow column (GE Healthcare) with a column volume of 1 mL and a gradient from 0 to 0.2 M NaCl in 50 mM Tris pH 7.0. The Coomassie-stained reducing SDS gel shows the (FT) column flow through, (WF) wash fraction, (1-8) elution fractions.

Figure S3. UV-Vis Spectrum of EGF-mCherry purified from E. coli after cytosolic expression.

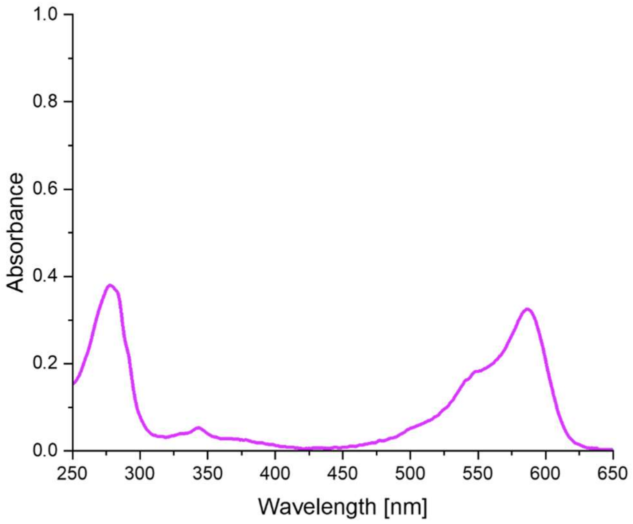

Figure S3. UV-Vis Spectrum of EGF-mCherry purified from *E. coli* after cytosolic expression. UV-Vis spectrum of EGF-mCherry at pH 7.4. Cytosolic expression in *E. coli* BL21(DE3) followed by IMAC purification was performed according to Feiner et al. [1].

Figure S4. pH-dependent fluorescence of CoGFP_V0 and EGF-CoGFP_V0 measured by a fluorescence plate reader.

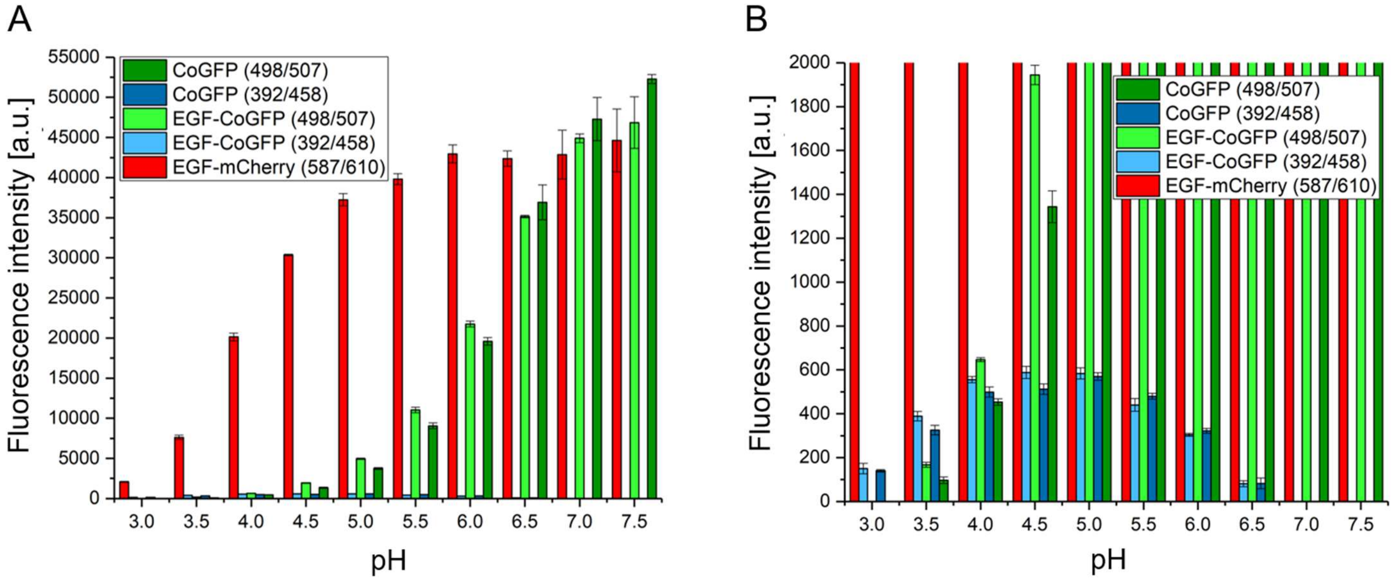

Figure S4. pH-dependent fluorescence of CoGFP_V0 and EGF-CoGFP_V0 measured by a fluorescence plate reader. pH titrations were performed in 0.1 M citric acid, 0.2 M Na_2HPO_4 for CoGFP_V0, EGF-CoGFP_V0 and EGF_mCherry to measure pH sensitivity of the fluorescence in a 96-well plate using the plate reader (POLARstar Galaxy, BMG LABTECH). The legend shows the excitation and emission maxima of each fluorescence in parentheses. The following filter sets were used: ex. 485-P, em. 520-P (CoGFP_V0, green), ex. 380-10, em. 450-10 (CoGFP-V0, blue), and ex. A-570, em. 645-10 (mCherry, red). Proteins and coloring are mentioned in parentheses, respectively. Calculated errors for each data point are the standard derivation of three technical replicates. Images show the bar plot with two different scalings to see (**A**) the green and red fluorescence of CoGFP_V0 and mCherry, and (**B**) the blue fluorescence of CoGFP_V0.

Figure S5. pH-dependent 3D fluorescence spectra of CoGFP_V0 and EGF-CoGFP_V0.

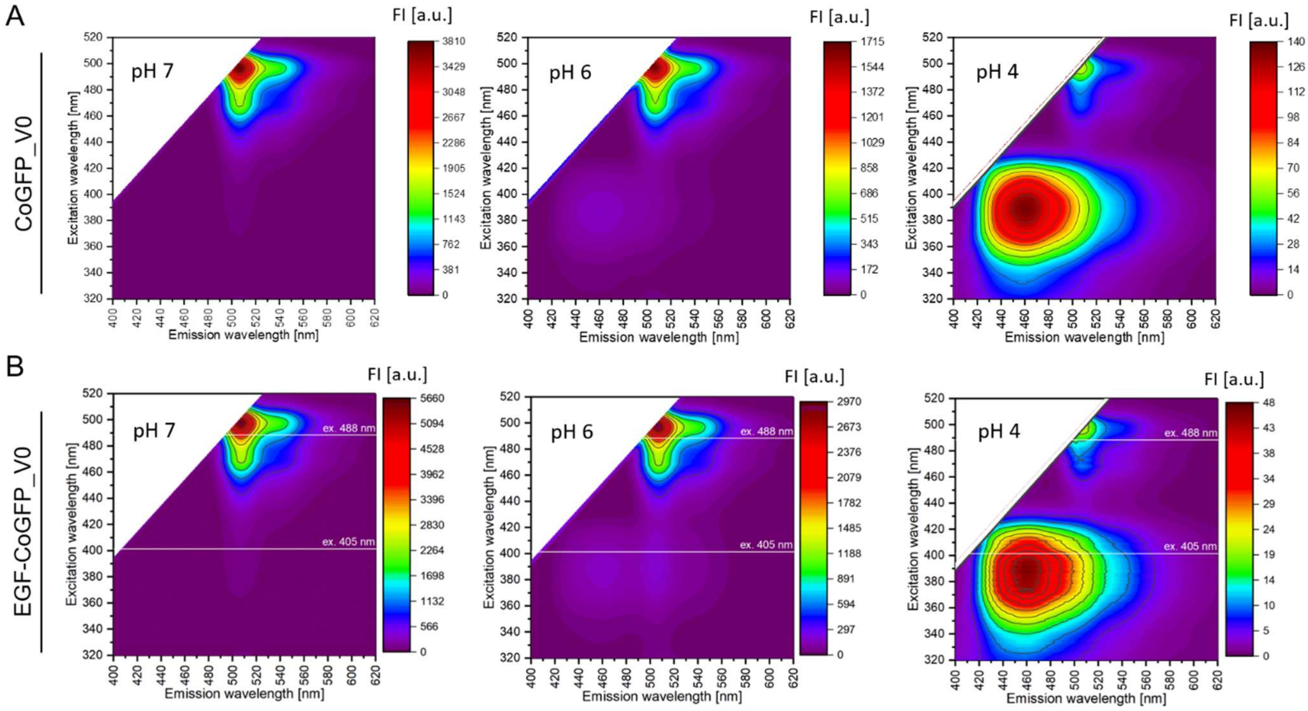

Figure S5. pH-dependent 3D fluorescence spectra of CoGFP_V0 and EGF-CoGFP_V0. 3D fluorescence spectra of (**A**) CoGFP_V0 and (**B**) EGF-CoGFP_V0 in 0.1 M citric acid, 0.2 M Na_2HPO_4 at pH 7, 6, and 4 were measured in a cuvette using a spectrofluorometer (1 nm ex. and 0.2 nm em. step scan). Common suitable excitation wavelengths 405 nm and 488 nm are marked in white. OriginPro was used to plot the 3D fluorescence spectra with absolute fluorescence intensities. Aggregation of CoGFP_V0 in solution at pH 4 contributes to lower fluorescence intensity during measurement in a cuvette. Fluorescence of EGF-CoGFP_V0 decreases more compared to CoGFP_V0 upon acidification probably due to increased aggregation tendency of the EGF part. For EGF-CoGFP_V0 at pH 4, the excitation at 405 nm instead of 388 nm leads to 78 % reduction in absorbance and concomitantly in emission at the maximum of 461 nm.

Figure S6. Live-cell imaging of A431 cells incubated with EGF-CoGFP_V0.

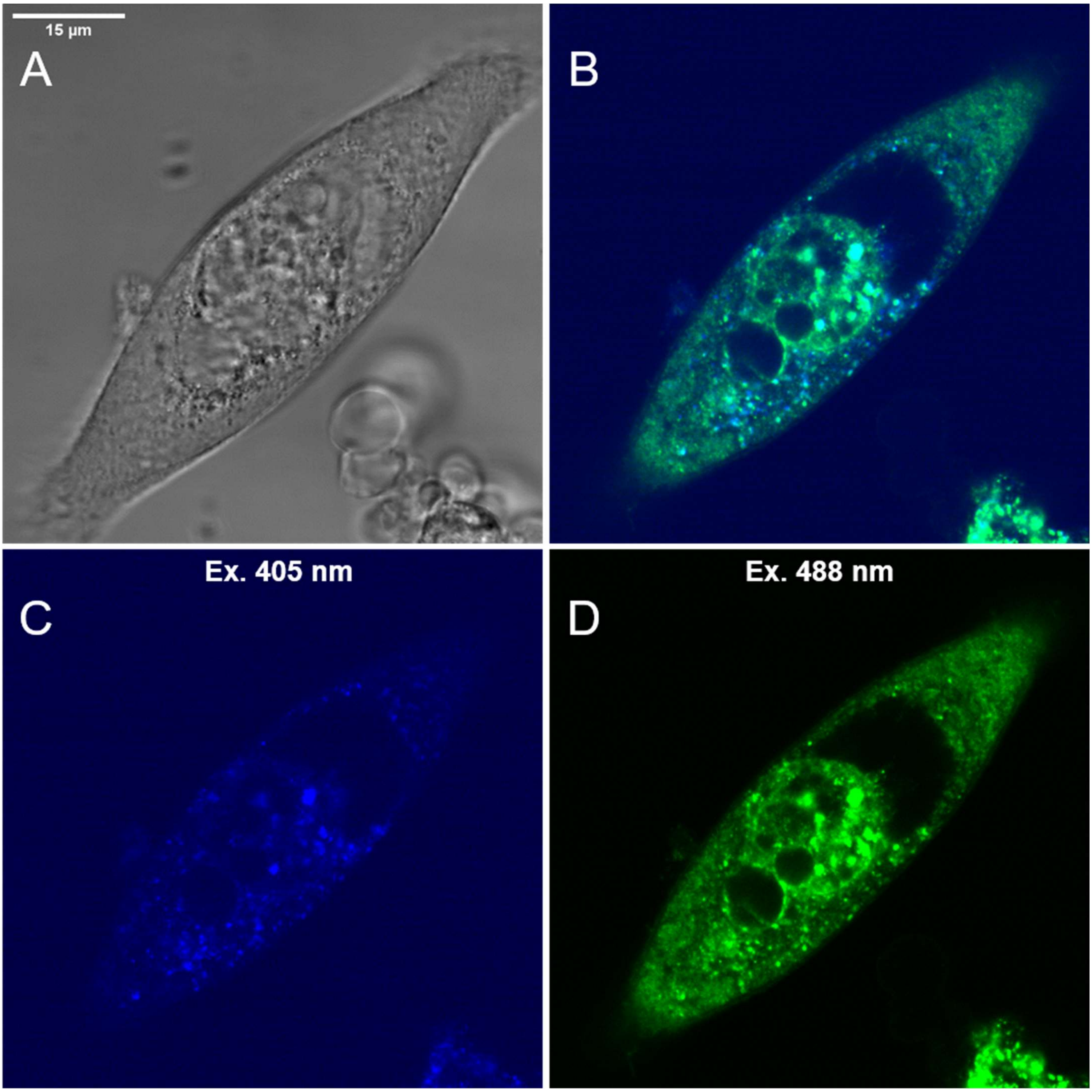

Figure S6. Live-cell imaging of A431 cells incubated with EGF-CoGFP_V0. A431 cells expressing high levels of EGFR were incubated with 100 nM EGF-CoGFP_V0 for 6 h at 37°C and 5 % CO_2, washed, further incubated for 2 h at 37°C and imaged using confocal fluorescence microscopy (LSM 780, Zeiss) with the objective LCI Plan-Neofluar 63×/1.3 Imm Corr DIC, MBS 405 and 488, laser 405 nm (2 % power, 1.59 Airy unit) with 410-597 nm emission detection, and laser 488 nm (2 % power, 1.51 Airy unit) with 491-597 emission detection. Frame scan modus and the 32-ch GaAsP detector was used for both channels. (**A**) Scale bar in the upper left corner of the bright-field image represents 15 µm. (**B**) Overlay of fluorescence images excited at 405 and 488 nm, respectively. (**C**) Fluorescence emission at 410-597 nm upon 405 nm excitation shows a high background fluorescence. (**D**) Fluorescence emission at 491-597 nm upon 488 nm excitation.

Figure S7. Expression of EGF-CoGFP-mTagBFP2 in the cytosol of E. coli.

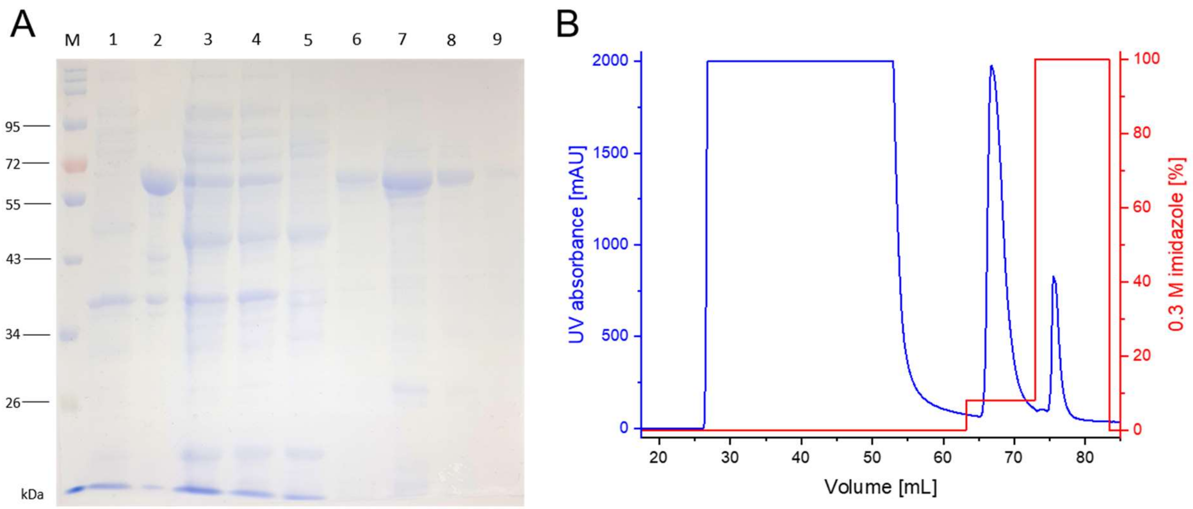

Figure S7. Expression of EGF-CoGFP-mTagBFP2 in the cytosol of *E. coli*. (**A**) Coomassie-stained reducing SDS gel after IMAC purification of EGF-CoGFP-mTagBFP2 in the cytosol of *E. coli* Origami B(DE3). (1) Cell lysate before IPTG induction, (2) insoluble fraction, (3) soluble fraction, (4) column flow through, (5) wash fraction, (6-9) elution fractions, (M) molecular marker. (**B**) UV absorbance (blue) and conductivity (red) of IMAC (Ni-NTA) for purification of EGF-CoGFP-mTagBFP2.

Table S1. List of identified proteins from MS/MS data evaluation.

Table S1. List of identified proteins from MS/MS data evaluation. Gel bands **B1**-**B6** from the SDS-PAGE (Fig. 1C) were analyzed by nanoLC-ESI-MS/MS. The two *E. coli* proteins with the highest Sequest HT Score are listed with additional information on coverage, number of peptides and findings (PSMs) in the sample, molecular weight and calculated pI.

Gel band	Description (Accession No.)	Coverage [%]	# Peptides	# PSMs	MW [kDa]	pI	Score
**B1**	ArnA (A0A140N587)	71	72	1336	74.2	6.87	3963.68
	GlmS (A0A140NE25)	58	37	123	66.9	5.87	526.94
**B2**	GlmS (A0A140NE25)	91	76	5567	66.9	5.87	26092.45
	ArnA (A0A140N587)	73	60	679	74.2	6.87	2918.14
**B3**	EGF-CoGFP-mTagBFP2	77	49	755	59.4	6.32	1787.45
	GlmS (A0A140N899)	58	31	87	55.7	5.76	204.71
**B4**	EGF-CoGFP-mTagBFP2	90	66	2233	59.4	6.32	8625.74
	ArnA (A0A140N587)	66	36	58	74.2	6.87	226.29
**B5**	Crp (A0A140N3D6)	98	32	1723	23.6	8.25	4544.72
	EGF-CoGFP-mTagBFP2	59	33	263	59.4	6.32	704.82
**B6**	Fur (A0A140NE13)	100	24	910	16.8	6.11	2896.62
	Rsd (A0A140SS61)	56	10	81	18.2	6.02	201.40

Table S2. List of identified peptide groups.

Table S2. List of identified peptide groups. EGF-CoGFP-mTagBFP2 was identified in the gel bands **B3** and **B4** from the SDS-PAGE (Fig. 1C) via nanoLC-ESI-MS/MS. The peptide groups are listed according to the sequence position with additional information on modifications and missed trypsin cleavages. The number of findings (PSMs) for each peptide are shown to compare the quantitative coverage of EGF-CoGFP-mTagBFP2 in the samples **B3** and **B4**. The modified precursor peptides [R].VIEGDPLPFSFDILSVAFQ.[Y] (position 109-128) and [K].VVEGGPLPFAFD-ILATSFL.[Y] (position 334-353) (Fig. S8) with C-terminal amidation (N(1) H(1) O(-1), Δmass= -0.984 Da) were found in sample **B4**. These chromophore-containing peptides might denature during the ionization process at 140°C as it was described by Gross et al. for DsRed [2]. The small modified chromophore containing peptides [Q].YGNR.[T] (position 129-132) and [L].YGSK.[T] (position 354-357) were not found.

# PSMs for gel band					
**B3**	**B4**	**Position**	**Peptide Sequence**	**Modification**	**Missed Cleavages**
	4	**1-29**	[-].MNSDSECPLSHDGYCLHDGVCMYI EALDK.[Y]	3xCarbamidomethyl [C7; C15; C21]	0
	2	**1-29**	[-].MNSDSECPLSHDGYCLHDGVCMYI EALDK.[Y]	3xCarbamidomethyl [C7; C15; C21]; 1xOxidation [M1]	0
	4	**1-42**	[-].MNSDSECPLSHDGYCLHDGVCMYI EALDKYACNCVVGYIGER.[C]	5xCarbamidomethyl [C7; C15; C21; C32; C34]	1
	1	**1-42**	[-].MNSDSECPLSHDGYCLHDGVCMYI EALDKYACNCVVGYIGER.[C]	5xCarbamidomethyl [C7; C15; C21; C32; C34]; 1xOxidation [M1]	1
6	20	**30-42**	[K].YACNCVVGYIGER.[C]	2xCarbamidomethyl [C3; C5]	0
1	1	**43-49**	[R].CQYRDLK.[W]	1xCarbamidomethyl [C1]	1
	2	**43-54**	[R].CQYRDLKWWELR.[G]	1xCarbamidomethyl [C1]	2
15	70	**47-54**	[R].DLKWWELR.[G]		1
	6	**55-105**	[R].GGGSGGGSSIPENSGLTEEMPAQM NLEGVVNGHAFSMEGIGGGNILTGIQ K.[L]		0
	7	**55-105**	[R].GGGSGGGSSIPENSGLTEEMPAQM NLEGVVNGHAFSMEGIGGGNILTGIQ K.[L]	1xOxidation [M]	0
	7	**55-105**	[R].GGGSGGGSSIPENSGLTEEMPAQM NLEGVVNGHAFSMEGIGGGNILTGIQ K.[L]	2xOxidation [M24; M]	0
	3	**55-105**	[R].GGGSGGGSSIPENSGLTEEMPAQM NLEGVVNGHAFSMEGIGGGNILTGIQ K.[L]	3xOxidation [M20; M24; M37]	0
1		**110-132**	[R].VIEGDPLPFSFDILSVAFQYGNR.[T]		0
33	33	**133-140**	[R].TYTSYPAK.[I]		0
	1	**133-157**	[R].TYTSYPAKIPDYFVQSFPEGFTFER.[T]		1
10	65	**141-157**	[K].IPDYFVQSFPEGFTFER.[T]		0

13	17	**158-168**	[R].TLSFEDGAIVK.[V]		0
1	74	**158-179**	[R].TLSFEDGAIVKVESDISIEDGK.[F]		1
3	156	**158-183**	[R].TLSFEDGAIVKVESDISIEDGKFVGK.[I]		2
2	4	**169-179**	[K].VESDISIEDGK.[F]		0
10	28	**169-183**	[K].VESDISIEDGKFVGK.[I]		1
9	25	**184-199**	[K].IKYNGEGFPEDGPVMK.[K]		1
8	9	**184-199**	[K].IKYNGEGFPEDGPVMK.[K]	1xOxidation [M15]	1
10	9	**184-200**	[K].IKYNGEGFPEDGPVMKK.[E]	1xOxidation [M15]	2
7	11	**184-200**	[K].IKYNGEGFPEDGPVMKK.[E]		2
6	22	**186-199**	[K].YNGEGFPEDGPVMK.[K]		0
7	9	**186-199**	[K].YNGEGFPEDGPVMK.[K]	1xOxidation [M13]	0
4	5	**186-200**	[K].YNGEGFPEDGPVMKK.[E]		1
6	5	**186-200**	[K].YNGEGFPEDGPVMKK.[E]	1xOxidation [M13]	1
	6	**200-228**	[K].KEVTKLEPSSESMYVSDGTLVGEVVLSYK.[T]		2
	2	**200-228**	[K].KEVTKLEPSSESMYVSDGTLVGEVVLSYK.[T]	1xOxidation [M13]	2
	53	**201-228**	[K].EVTKLEPSSESMYVSDGTLVGEVVLSYK.[T]		1
	67	**205-228**	[K].LEPSSESMYVSDGTLVGEVVLSYK.[T]		0
1	17	**205-228**	[K].LEPSSESMYVSDGTLVGEVVLSYK.[T]	1xOxidation [M8]	0
	2	**205-239**	[K].LEPSSESMYVSDGTLVGEVVLSYKTQSTHYTCHMK.[T]	1xCarbamidomethyl [C32]	1
7	15	**229-239**	[K].TQSTHYTCHMK.[T]	1xCarbamidomethyl [C8]	0
11	22	**229-239**	[K].TQSTHYTCHMK.[T]	1xCarbamidomethyl [C8]; 1xOxidation [M10]	0
	1	**240-253**	[K].TIYRSKKPVENLPK.[F]		2
13	46	**244-253**	[R].SKKPVENLPK.[F]		1
7	24	**246-253**	[K].KPVENLPK.[F]		0
10	18	**254-260**	[K].FHYVHHR.[L]		0
4	7	**254-263**	[K].FHYVHHRLEK.[K]		1
	2	**254-264**	[K].FHYVHHRLEKK.[I]		2
6	52	**264-286**	[K].KIVEEGYYYEQHETAIAKPGSLR.[S]		1
5	65	**265-286**	[K].IVEEGYYYEQHETAIAKPGSLR.[S]		0
4	5	**287-296**	[R].SVSKGEELIK.[E]		1
6	16	**287-302**	[R].SVSKGEELIKENMHMK.[L]		2
19	31	**287-302**	[R].SVSKGEELIKENMHMK.[L]	1xOxidation [M]	2
13	14	**287-302**	[R].SVSKGEELIKENMHMK.[L]	2xOxidation [M13; M15]	2

4	8	**291-302**	[K].GEELIKENMHMK.[L]		1
6	11	**291-302**	[K].GEELIKENMHMK.[L]	1xOxidation [M]	1
3	3	**291-302**	[K].GEELIKENMHMK.[L]	2xOxidation [M9; M11]	1
1	4	**297-302**	[K].ENMHMK.[L]		0
1	1	**297-302**	[K].ENMHMK.[L]	1xOxidation [M3]	0
11	43	**303-315**	[K].LYMEGTVDNHHFK.[C]		0
16	20	**303-315**	[K].LYMEGTVDNHHFK.[C]	1xOxidation [M3]	0
11	17	**316-332**	[K].CTSEGEGKPYEGTQTMR.[I]	1xCarbamidomethyl [C1]; 1xOxidation [M16]	0
6	13	**316-332**	[K].CTSEGEGKPYEGTQTMR.[I]	1xCarbamidomethyl [C1]	0
26	164	**358-371**	[K].TFINHTQGIPDFFK.[Q]		0
18	3	**358-382**	[K].TFINHTQGIPDFFKQSFPEGFTWER.[V]		1
	78	**372-382**	[K].QSFPEGFTWER.[V]		0
2	84	**383-410**	[R].VTTYEDGGVLTATQDTSLQDGCLIY NVK.[I]	1xCarbamidomethyl [C22]	0
	1	**383-412**	[R].VTTYEDGGVLTATQDTSLQDGCLIY NVKIR.[G]	1xCarbamidomethyl [C22]	1
4	5	**411-425**	[K].IRGVNFTSNGPVMQK.[K]		1
4	3	**411-425**	[K].IRGVNFTSNGPVMQK.[K]	1xOxidation [M13]	1
	1	**411-426**	[K].IRGVNFTSNGPVMQKK.[T]		2
5	24	**413-425**	[R].GVNFTSNGPVMQK.[K]		0
18	7	**413-425**	[R].GVNFTSNGPVMQK.[K]	1xOxidation [M11]	0
3	4	**413-426**	[R].GVNFTSNGPVMQKK.[T]		1
4	4	**413-426**	[R].GVNFTSNGPVMQKK.[T]	1xOxidation [M11]	1
1	15	**426-447**	[K].KTLGWEAFTETLYPADGGLEGR.[N]		1
	2	**426-453**	[K].KTLGWEAFTETLYPADGGLEGRND MALK.[L]		2
	2	**426-453**	[K].KTLGWEAFTETLYPADGGLEGRND MALK.[L]	1xOxidation [M25]	2
2	83	**427-447**	[K].TLGWEAFTETLYPADGGLEGR.[N]		0
	3	**427-453**	[K].TLGWEAFTETLYPADGGLEGRND MALK.[L]	1xOxidation [M24]	1
	3	**427-453**	[K].TLGWEAFTETLYPADGGLEGRND MALK.[L]		1
14	19	**448-453**	[R].NDMALK.[L]	1xOxidation [M3]	0
	4	**448-465**	[R].NDMALKLVGGSHLIANAK.[T]		1
	2	**448-465**	[R].NDMALKLVGGSHLIANAK.[T]	1xOxidation [M3]	1
37	128	**454-465**	[K].LVGGSHLIANAK.[T]		0
2	2	**454-469**	[K].LVGGSHLIANAKTTYR.[S]		1

	1	**466-475**	[K].TTYRSKKPAK.[N]		2
5	6	**476-488**	[K].NLKMPGVYYVDYR.[L]		1
10	6	**476-488**	[K].NLKMPGVYYVDYR.[L]	1xOxidation [M4]	1
2	1	**476-491**	[K].NLKMPGVYYVDYRLER.[I]	1xOxidation [M4]	2
	5	**476-491**	[K].NLKMPGVYYVDYRLER.[I]		2
74	95	**479-488**	[K].MPGVYYVDYR.[L]		0
26	14	**479-488**	[K].MPGVYYVDYR.[L]	1xOxidation [M1]	0
3	7	**479-491**	[K].MPGVYYVDYRLER.[I]		1
4	5	**479-491**	[K].MPGVYYVDYRLER.[I]	1xOxidation [M1]	1
43	111	**492-510**	[R].IKEANNETYVEQHEVAVAR.[Y]		1
	1	**492-517**	[R].IKEANNETYVEQHEVAVARYCDLP SK.[L]	1xCarbamidomethyl [C21]	2
3	4	**494-510**	[K].EANNETYVEQHEVAVAR.[Y]		0
72	70	**511-517**	[R].YCDLPSK.[L]	1xCarbamidomethyl [C2]	0
1	1	**511-521**	[R].YCDLPSKLGHK.[L]	1xCarbamidomethyl [C2]	1
55	80	**522-529**	[K].LNHHHHHH.[-]		0

Figure S8. Protein sequence of EGF-CoGFP-mTagBFP2.

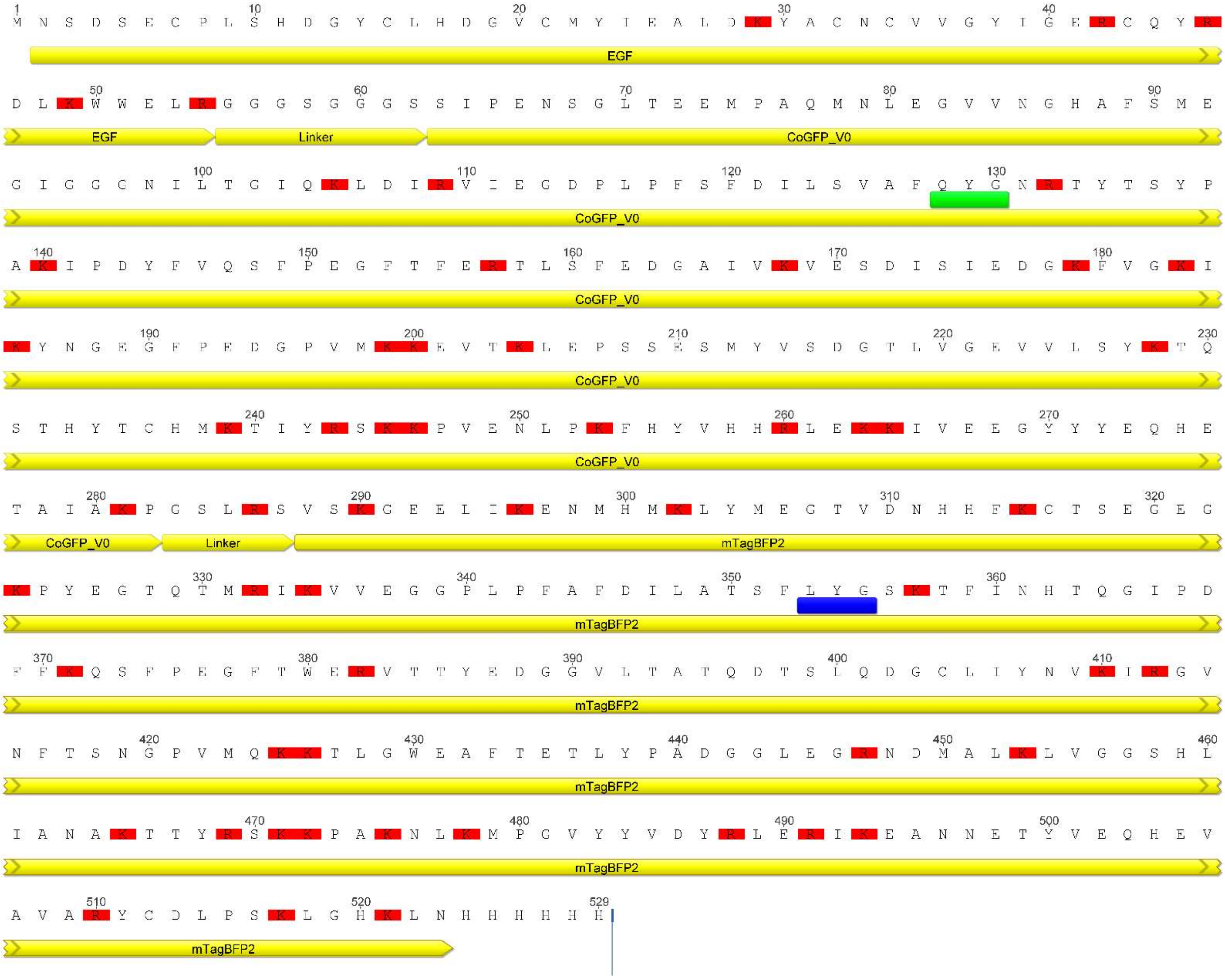

Figure S8. Protein sequence of EGF-CoGFP-mTagBFP2. Sequences of EGF, CoGFP_V0, mTagBFP2 and linkers are annotated with yellow bars. Chromophore tripeptides are annotated for CoGFP_V0 (green) and mTagBFP2 (blue). All potential trypsin cleavage sites (arginine, lysine) are marked in red.

Figure S9. Multiple protein sequence alignment of fluorescent proteins.

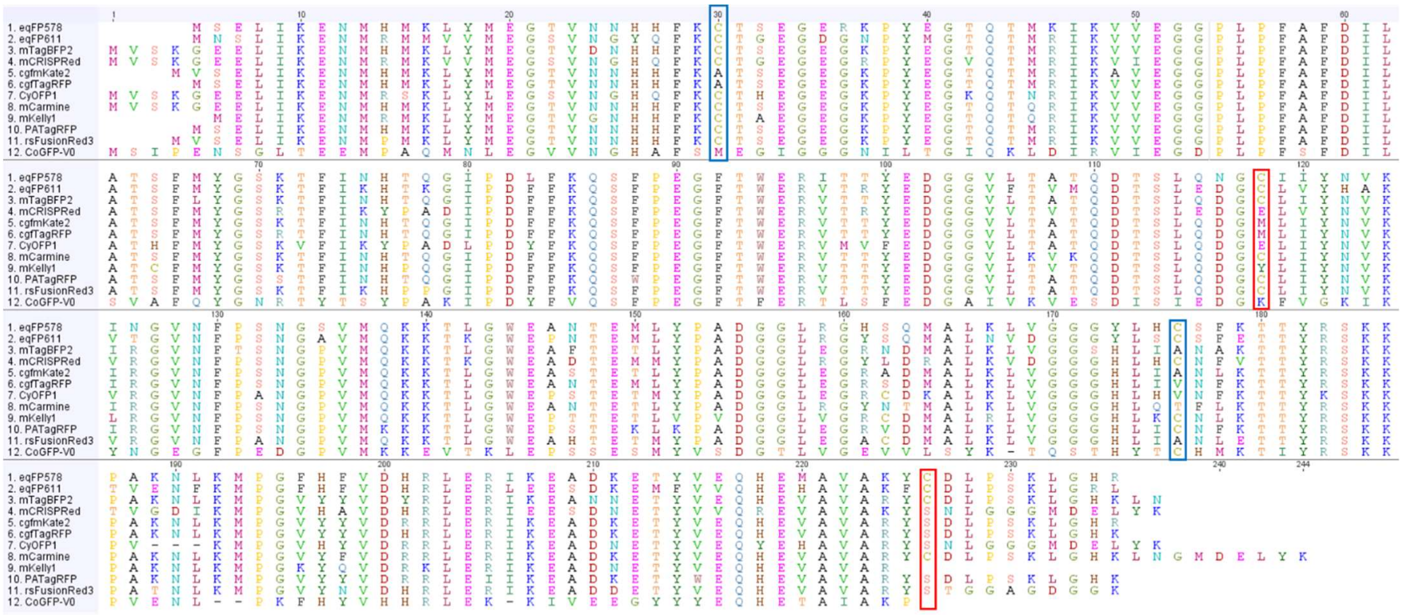

Figure S9. Multiple protein sequence alignment of fluorescent proteins. eqFP578 [3] and eqFP611 [4] both originate from *Entacmaea quadricolor*. eqFP611 is the parent protein of mCRISPRed [5]. eqFP578 is the parent protein of mTagBFP2 [6], cgfmKate2 [7], cgfTagRFP [7], CyOFP1 [8], mCarmine [9], mKelly1 [10], PATagRFP [11] and rsFusionRed3 [12]. The alignment covers all *E. quadricolor* fluorescent proteins that have any cysteine substitutions at the amino acid positions 30, 118, 176 or 226 (numbering according to sequence alignment). Thiol groups from C30 and C176 (blue boxes) are facing to the inside and from C118 and C226 (red boxes) to the outside of the β-barrel structure. The protein sequences were taken from the fluorescent protein database FPbase [13]. For comparison CoGFP_V0 [14] from *Cavernularia obesa* is aligned as well. Global alignment was performed using the Geneious alignment tool with free end gaps (Geneious 9.0.5).

Figure S10. Expression of EGF-CoGFP-mCRISPRed in the cytosol of E. coli.

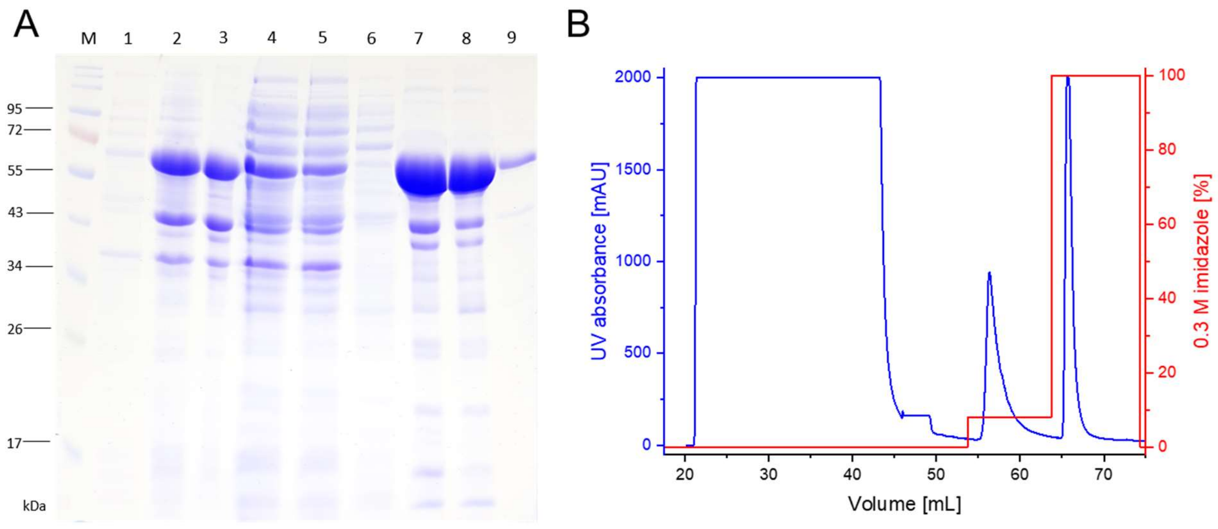

Figure S10. Expression of EGF-CoGFP-mCRISPRed in the cytosol of *E. coli*. (**A**) Coomassie-stained reducing SDS gel after IMAC purification of EGF-CoGFP-mCRISPRed, which was expressed in the cytosol of *E. coli* BL21(DE3). (1) Cell lysate before IPTG induction, (2) cell lysate after IPTG induction (3) insoluble fraction, (4) soluble fraction, (5) column flow through, (6) wash fraction, (7-9) elution fractions, (M) molecular marker. (**B**) UV absorbance (blue) and conductivity (red) of IMAC (Ni-NTA) for purification of EGF-CoGFP-mCRISPRed.

Figure S11. Binding specificity of EGF-CoGFP_V0 and EGF-CoGFP-mCRISPRed towards EGFR.

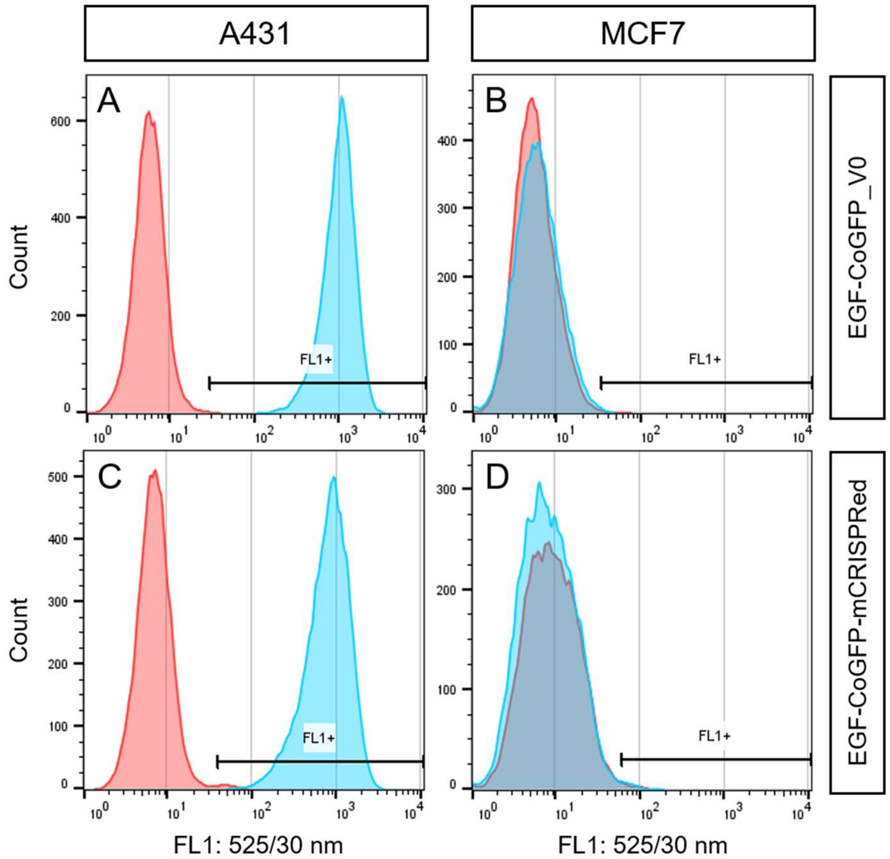

Figure S11. Binding specificity of EGF-CoGFP_V0 and EGF-CoGFP-mCRISPRed towards EGFR. Flow cytometry analysis showed that EGF-CoGFP_V0 (blue population) selectively bound to (**A**) A431 cells expressing high level of EGFR, but not to (**B**) MCF7 cells expressing low level of EGFR in comparison to untreated cells (red population). The same binding behavior was observed for the tandem fluorescent construct EGF-CoGFP-mCRISPRed (blue population), which selectively bound to (**C**) A431 cells, but not to (**D**) MCF7 cells in comparison to untreated cells (red population). 2×10^6 and 2×10^3 EGFR sites per cell for the A431 squamous cell carcinoma and the MCF7 breast cancer cells has been reported, respectively [15]. The binding behavior of these EGF fusion proteins are in agreement with the EGF-mCherry, which has been previously extensively characterized [1].

Figure S12. Expression of mTagBFP2 in the cytosol of E. coli.

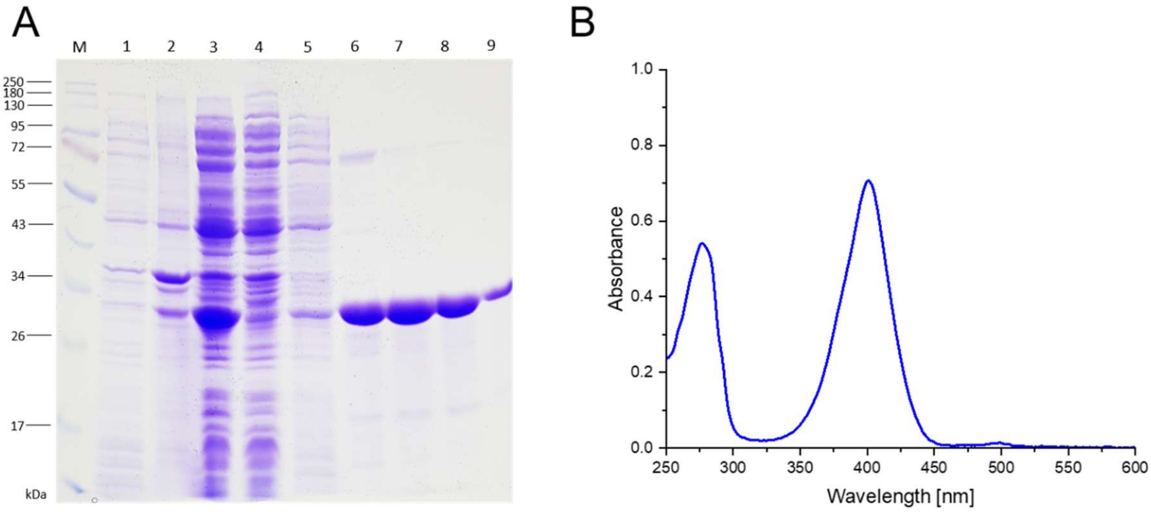

Figure S12. Expression of mTagBFP2 in the cytosol of *E. coli*. (**A**) Coomassie-stained reducing SDS gel after IMAC purification of mTagBFP2, which was expressed in the cytosol of *E. coli* BL21(DE3). (1) Cell lysate before IPTG induction, (2) insoluble fraction, (3) soluble fraction, (4) column flow through, (5) wash fraction, (6-9) elution fractions, (M) molecular marker. (**B**) UV-Vis spectrum of mTagBFP2 at pH 8.

Figure S13. Sequence alignment of different FRET linkers for tandem fluorescent proteins.

ECFP-Venus	EFVTAAGITLGMDELYK.....MVSKGEELFTGVVPILV
Cy11.5	EFVTAA-----------LE...-----EELFTGVVPILV
EGF-CoGFP-mTagBFP2	ETAIAKP..............MVSKGEELIKENMHMKL
EGF-CoGFP-Δ-mTagBFP2	ETAIAKP.........LE...-----EELIKENMHMKL
C5V	EFVTAAGITLGMDELYKSGLRS-VSKGEELFTGVVPILV
EGF-CoGFP-mTagBFP2	ETAIAKP.........GSLRS-VSKGEELIKENMHMKL
EGF-CoGFP-mCRISPRed	ETAIAKP.........GSLRS-VSKGEELIKENMRMKV

Figure S13. Sequence alignment of different FRET linkers for tandem fluorescent proteins. The pH-dependent spectral overlap of mTagBFP2 as a donor and CoGFP_V0 as an acceptor indicates a possible FRET pair. Tight concatenation of both fluorescent proteins to a chimeric protein according to Shimozono et al. resulted in poor folding of EGF-CoGFP-Δ-mTagBFP2 (data not shown) [16]. The chimeric protein Cy11.5 in Shimozono et al. consists of the fluorescent proteins ECFP and Venus. Therefore, the tandem fluorescent protein of the final EGF-CoGFP-mTagBFP2 construct was linked by five amino acids following the C5V design as FRET reference standard of Koushik

et al. (Koushik et al., 2006; Thaler et al., 2005). EGF-CoGFP-mCRISPRed containing the large Stokes-shift fluorescent protein was equivalently constructed. Legend: Deleted amino acid (-), no amino acid present (.), α-helix (cyan), β-sheet (yellow), linker (magenta).

Figure S14. pH-dependent 3D fluorescence spectra of mTagBFP2.

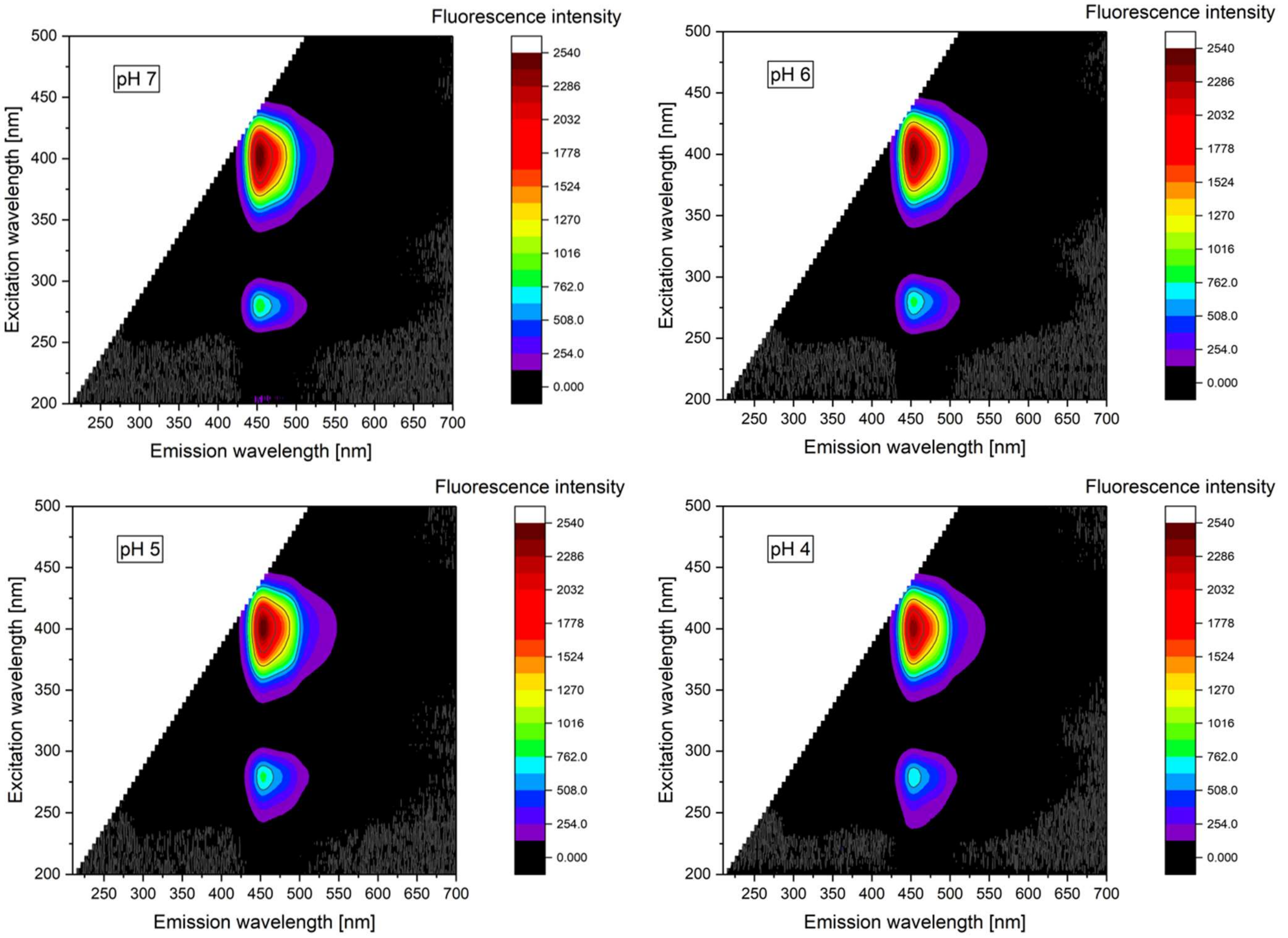

Figure S14. pH-dependent 3D fluorescence spectra of mTagBFP2. 3D fluorescence spectra of mTagBFP2 in citric acid, 0.2 M Na_2HPO_4 at pH 7, 6, 5 and 4 were measured using a spectrofluorometer. OriginPro was used to plot the 3D fluorescence spectra with absolute fluorescence intensities. All four plots share the same scaling of fluorescence intensity showing a constant blue fluorescence emission among all pH values.

Figure S15. Indirect determination of FRET efficiciency from 3D fluorescence spectra.

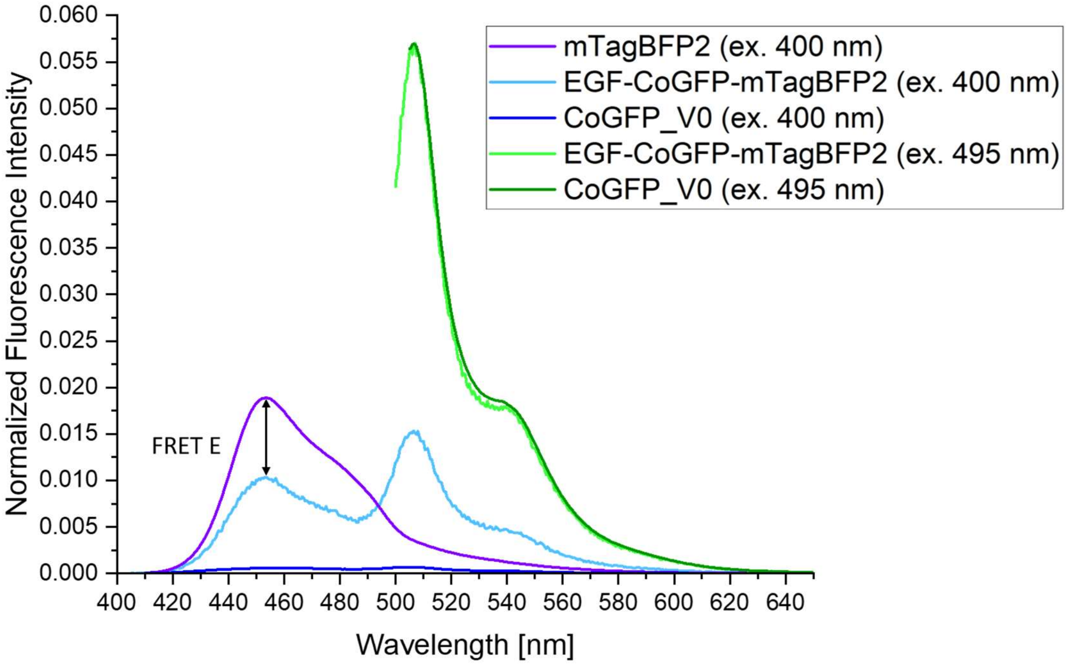

Figure S15. Indirect determination of FRET efficiency from 3D fluorescence spectra. The plot shows normalized emission spectra upon excitation (ex.) at 400 nm of mTagBFP2 (purple), EGF-CoGFP-mTagBFP2 (light blue) and CoGFP_V0 (dark blue) and upon excitation at 495 nm of EGF-CoGFP-mTagBFP2 (light green) and CoGFP_V0 (dark green). All spectra were extracted from 3D fluorescence spectra measured at pH 7 (Fig. 3, S5, S14). To determine the (low) contribution of CoGFP_V0 to the calculation of the FRET efficiency from the 3D spectra, the following calculations were done. First, the donor (mTagBFP2) and FRET pair (EGF-CoGFP-mTagBFP2) emission spectra upon excitation at 400 nm were normalized to unit area with $\int_0^\infty y(\lambda)d\lambda = 1$. Second, applying the resulting scaling factor also to the FRET pair emission upon 495 nm excitation as part of the 3D fluorescence spectra gave the light green spectrum. This provided an emission maximum for 495 nm excitation correlated with the normalized spectrum at 400 nm excitation. Third, the acceptor (CoGFP_V0) emission maximum upon 495 nm excitation was set to this correlated FRET emission maximum, which is a reasonable equivalent as there is no contribution of mTagBFP2 emission at 495 nm excitation (spectrum not shown) resulting in the dark green spectrum. Forth, the scaling factor obtained for the CoGFP_V0 adjustment at 495 excitation was applied to the CoGFP_V0 spectrum obtained at 400 nm excitation resulting in the dark blue spectrum. This showed that contributions of CoGFP_V0 emission upon 400 nm excitation was negligible within an acceptable margin of error for the FRET E determination. Finally, the emission ratio from donor and FRET pair (black arrow) at the maximum 453 nm (ex. 400 nm) was used to determine the FRET efficiency resulting in 45% at pH 7 which relates to an average distance of r= 5.7 nm according to Equation (3) solved for *r*. FRET efficiencies at pH 6 or pH 5 were 30% or 9%, respectively, when using the corresponding r_0 values and assuming a constant distance r upon pH shifts.

Table S3. Maturation times of fluorescent proteins from Entacmaea quadricolor.

Table S3. Maturation times for different fluorescent proteins originated from *Entacmaea quadricolor*.

**Protein**	**Temperature**	$t_{0.5}$	$t_{0.9}$	**Reference**
eqFP611	24.5°C	4.5 h	~ 12 h	[4,17]
mRuby	37°C	2.8 h	~ 8 h	[18]
mRuby2	37°C	2.5 h		[19]

mRuby3	37°C	2.2 h	5.7 h	[20]
mRuby3	32°C	4.1 h	12.1 h	[20]
mCRISPRed	37°C	5.9 h		[21]
mTagBFP2	37°C	12 min		[6]

Figure S16. Maturation assay of EGF-CoGFP-mCRISPRed.

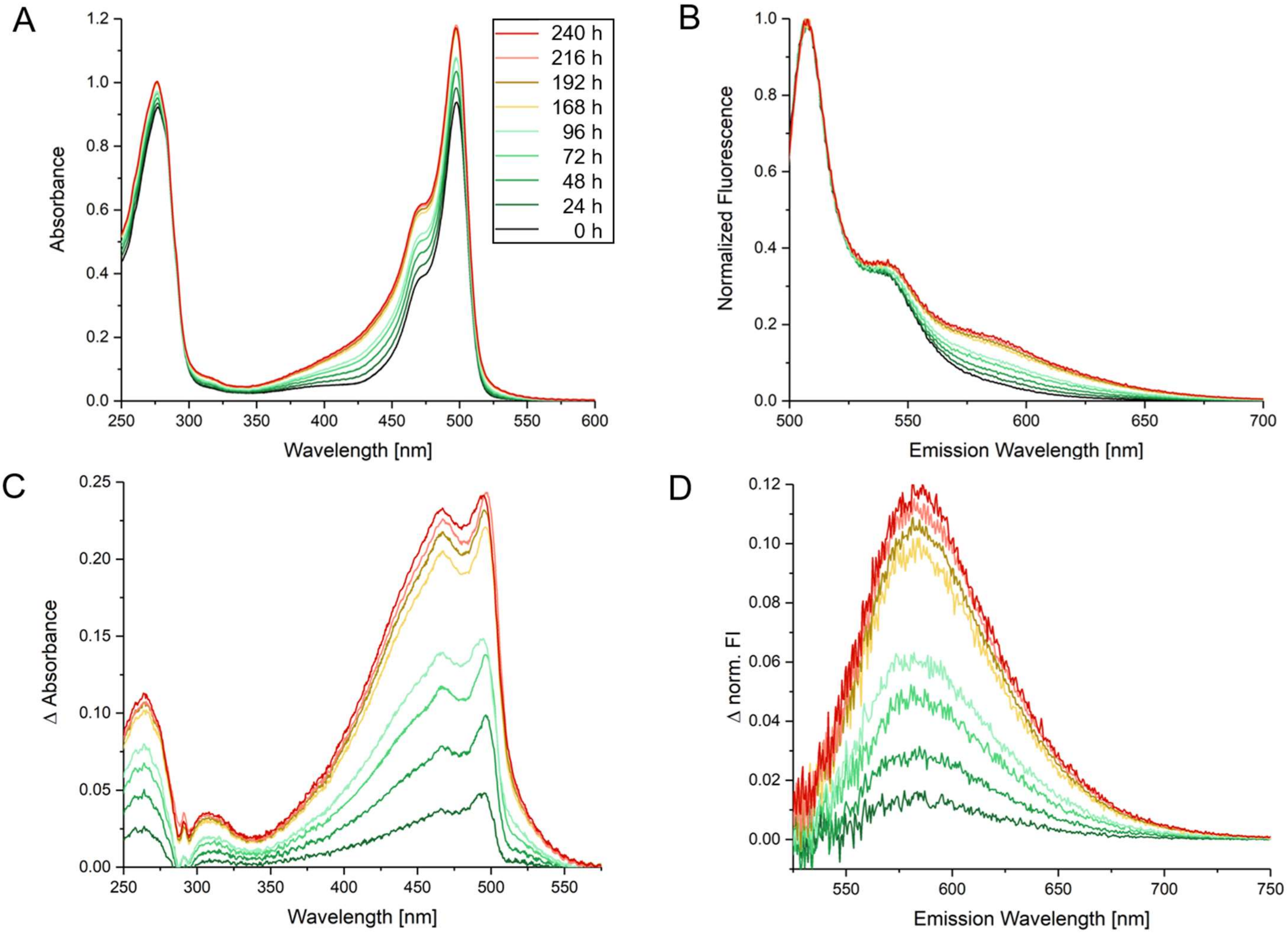

Figure S16. Maturation assay of EGF-CoGFP-mCRISPRed. Freshly thawed EGF-CoGFP-mCRISPRed stored in PBS (pH 7.4) at 4°C. (**A**) UV-Vis absorbance and (**B**) fluorescence emission at 485 nm excitation of the same protein sample was measured at the indicated time points at 20°C. The samples were stored at 4°C between each measurement. (**C**) Difference absorbance spectra and (**D**) difference spectra of the normalized fluorescence intensity show the maturation process over time.

Figure S17. Data acquisition for calibration curves of EGF-CoGFP-mTagBFP2 and EGF-CoGFP-mCRISPRed.

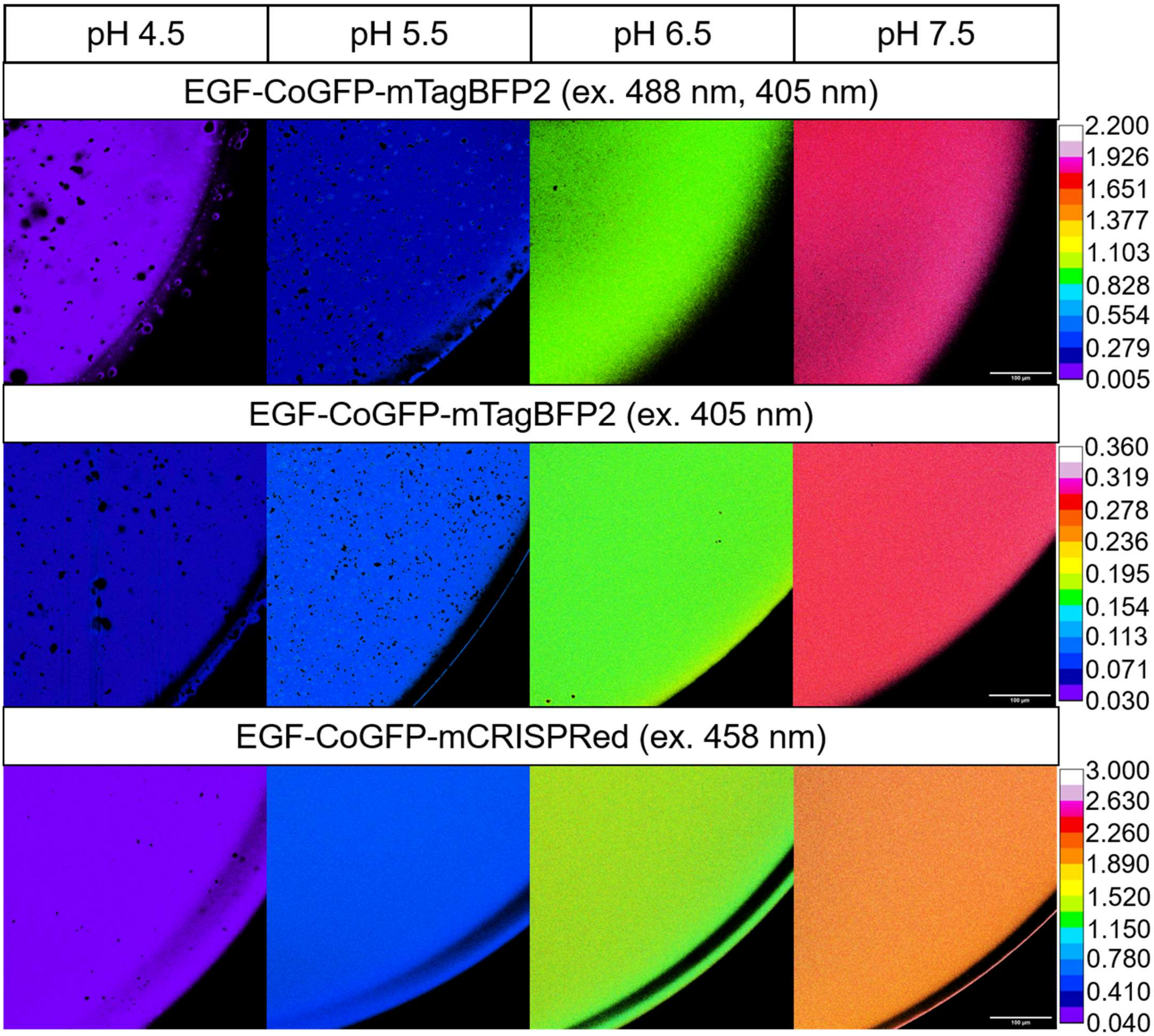

Figure S17. Data acquisition for calibration curves of EGF-CoGFP-mTagBFP2 and EGF-CoGFP-mCRISPRed. Titrated protein solutions of EGF-CoGFP-mTagBFP2 and EGF-CoGFP-mCRISPRed were imaged using confocal microscopy. Upper panel: Ratio 500 nm (ex. 488 nm) / 455 nm (ex. 405 nm) of EGF-CoGFP-mTagBFP2. Middle panel: 500 nm (ex. 405 nm) / 455 nm (ex. 405 nm) for EGF-CoGFP-mTagBFP2. Lower panel: 500 nm (ex. 458 nm) / 590 nm (ex. 458 nm) for EGF-CoGFP-mCRISPRed. Pixel-to-pixel intensity ratios were calculated using ImageJ software [22]. Oversaturated fluorescence intensities caused by protein aggregates or droplet margin were thresholded. For each pH value the mean ratio was calculated for the whole image area (NaN background). Ratios were pseudo colored according to the calibration bars at the right-hand side.

Table S4. Calculated variables for calibration curves of EGF-CoGFP-mTagBFP2 and EGF-CoGFP-mCRISPRed.

Table S4. Calculated variables for calibration curves of EGF-CoGFP-mTagBFP2 and EGF-CoGFP-mCRISPRed. Emission ratios at indicated excitation wavelength for EGF-CoGFP-mTagBFP2 and EGF-CoGFP-mCRISPRed were fitted using the OriginPro software to obtain calibration curves. The resulting ratiometric equilibrium *pK′*, the ratiometric offset R_0, the dynamic range R_f and the R^2 coefficient of determination for the regression are listed.

pH Sensor Molecule	Emission Ratio	Excitation	*pK′*	R_f	R_0	R^2
EGF-CoGFP-mTagBFP2	526 nm / 448 nm	488 nm / 405 nm	6.6	1046.3	0.002	0.969
EGF-CoGFP-mTagBFP2	500 nm / 455 nm	405 nm / 405 nm	6.4	5.7	0.054	0.989
EGF-CoGFP-mCRISPRed	508 nm / 659 nm	458 nm / 458 nm	6.1	29.6	0.074	0.996

Figure S18. Live-cell imaging of untreated cells for autofluorescence correction.

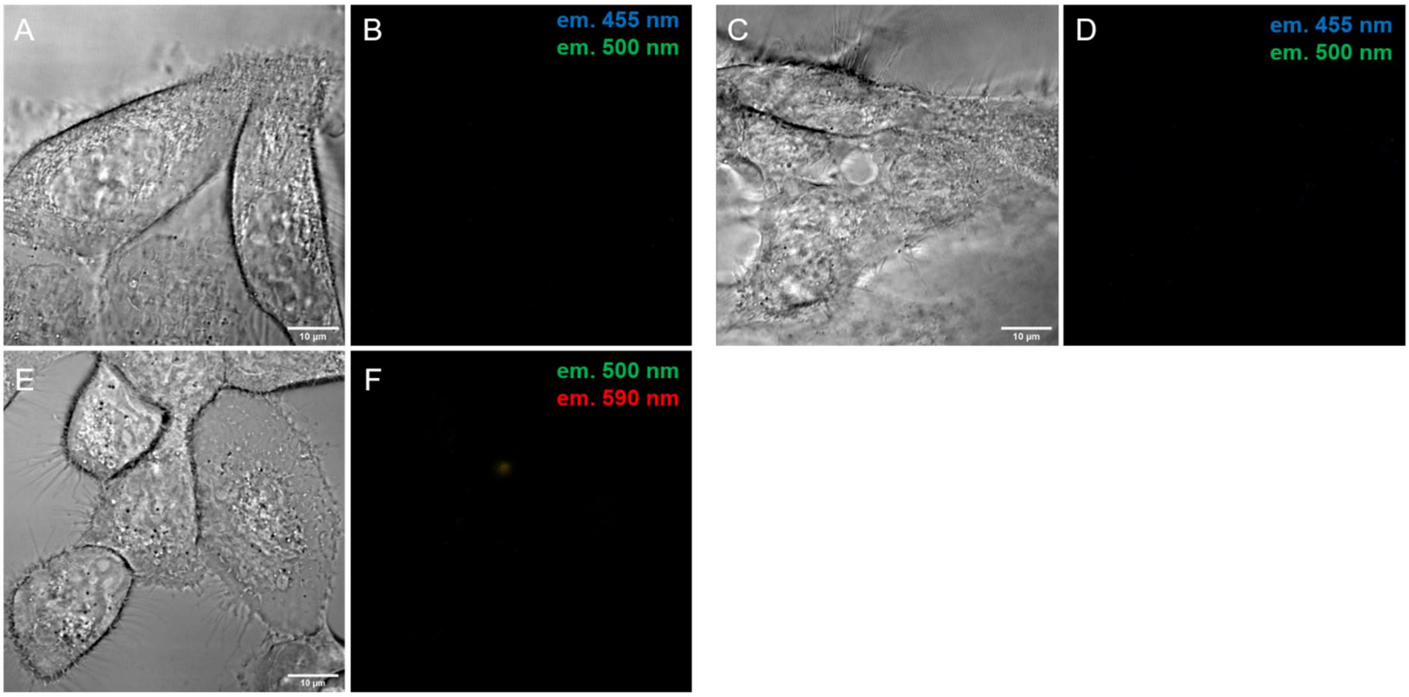

Figure S18. Live-cell imaging of untreated cells for autofluorescence correction. As a negative control untreated A431 cells were cultured at 37°C and imaged using confocal fluorescence microscopy. (**A**, **C**, **E**) Scale bars in the lower right corner of the bright-field images represent 10 µm. Fluorescent emission was detected at (**B**) 455 nm (ex. 405 nm), 500 nm (ex. 488 nm), (**D**) 455 nm and 500 nm (both ex. 405 nm), (**F**) 500 nm and 590 nm (both ex. 458 nm).

Figure S19. Intracellular pH-mapping using FRET properties of EGF-CoGFP-mTagBFP2.

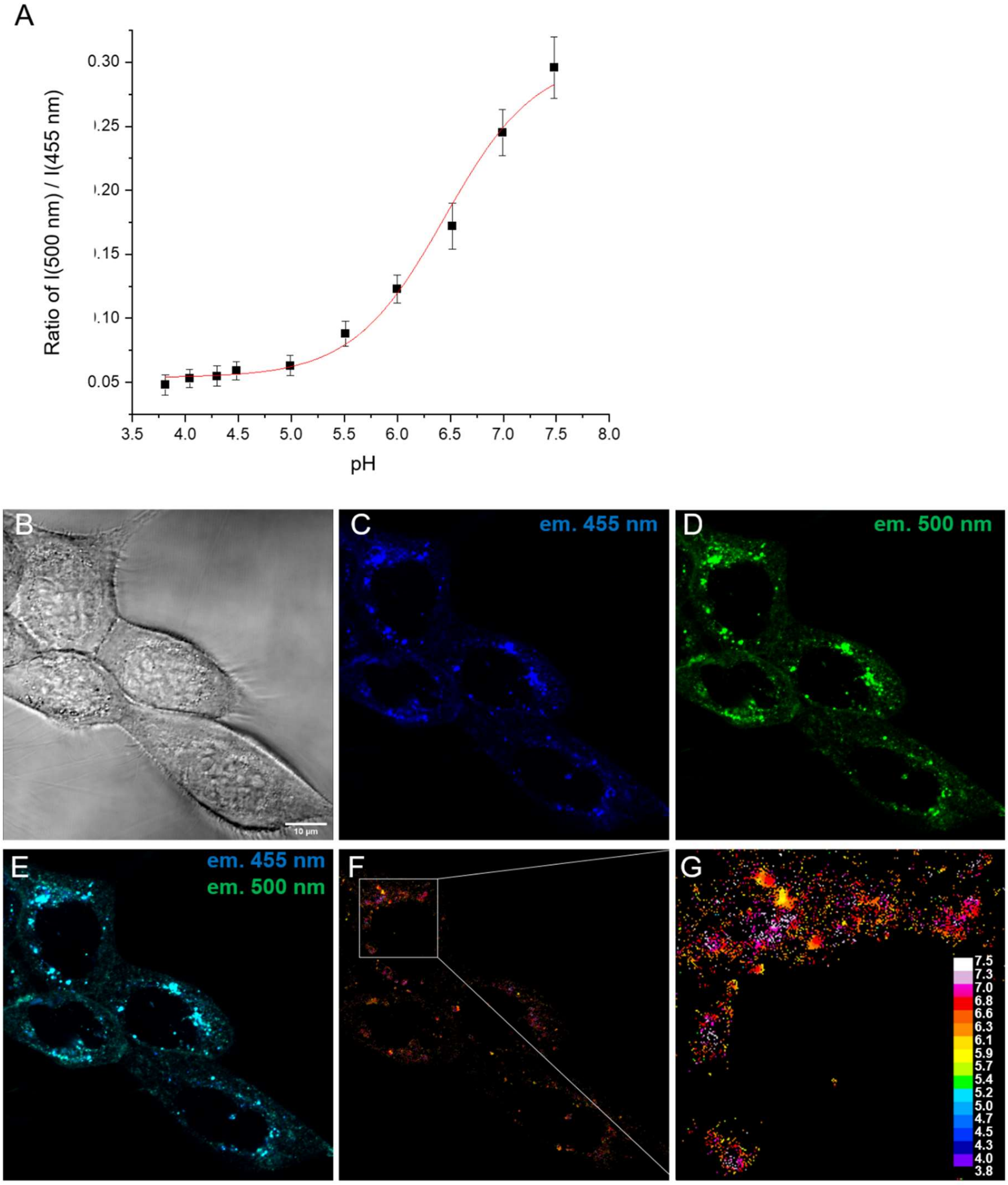

Figure S19. Intracellular pH-mapping using FRET properties of EGF-CoGFP-mTagBFP2. (**A**) Fluorescence measurements of titrated EGF-CoGFP-mTagBFP2 protein solutions were performed using confocal microscopy and mean values of emission ratios 500 nm (ex. 405 nm) / 455 nm (ex. 405 nm) were calculated. Mean values of ratios were obtained from processed images using ImageJ software [22]. Error bars represent the standard deviation. Data points were fitted using OriginPro software. A431 cells expressing high levels of EGFR were incubated with 100 nM EGF-CoGFP-mTagBFP2 for 10 min, washed, further incubated for 50 min at 37°C and imaged using confocal fluorescence microscopy. (**B**) Scale bar in the lower right corner of the bright-field image represents 10 µm. Single-wavelength excitation at 405 nm was used to obtain fluorescence images at (**C**) 500 nm (green) and (**D**) 455 nm (bue) emission wavelengths. Pixel-to-pixel intensity ratios (**E**) were calculated and converted with the corresponding calibration curve into pH values. (**F**-**G**) The resulting intracellular pH map was filtered for the median over a 2×2 pixel area and

pseudo colored according to the calibration bar. The white box indicates a selected area for (**G**) an image magnification.

Figure S20. Luminal pH-gradients of endosomal and lysosomal vesicles.

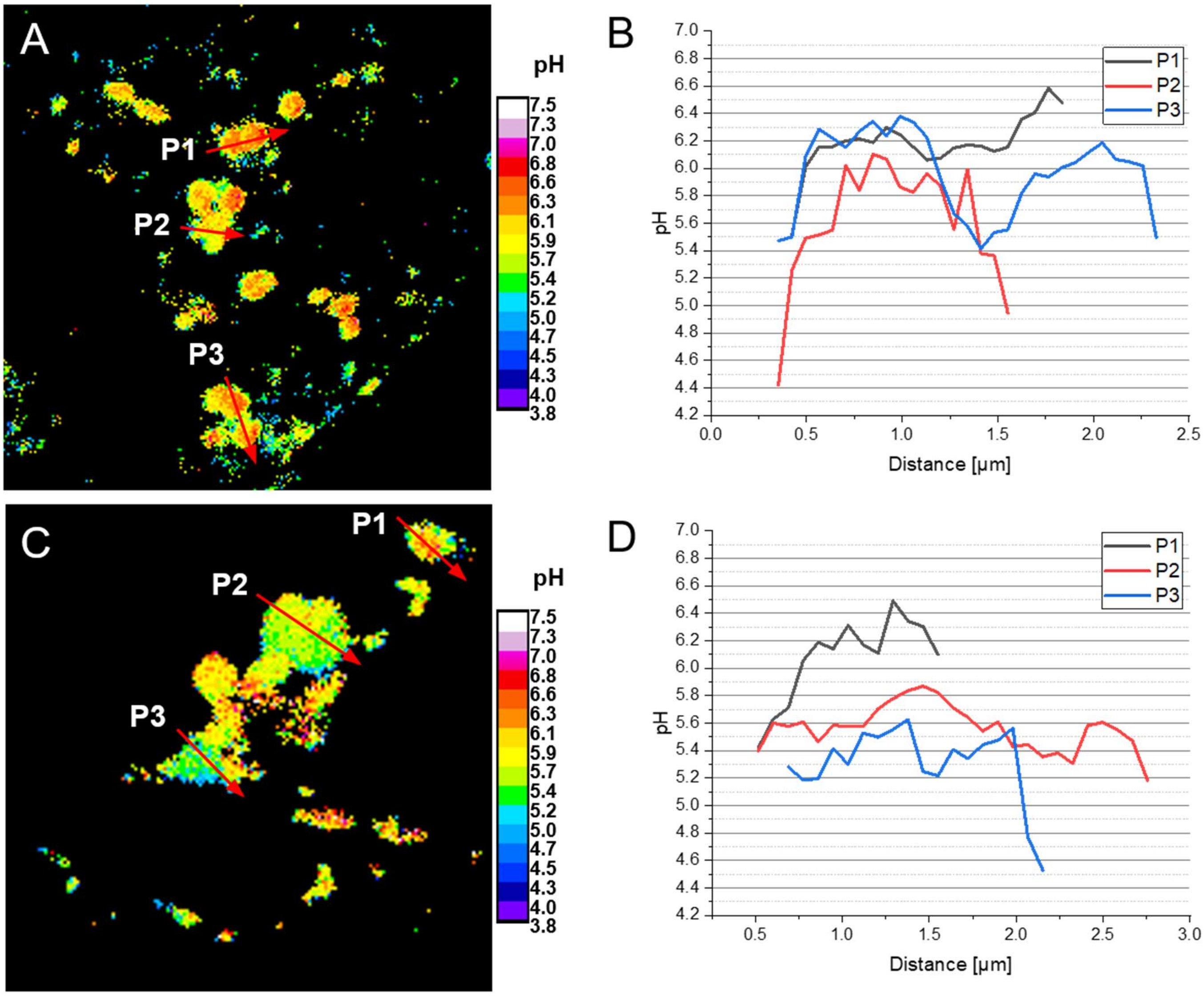

Figure S20. Luminal pH-gradients of endosomal and lysosomal vesicles. Intracellular pH maps and corresponding profile plots of (**A**, **B**) EGF-CoGFP-mTagBFP2 (emission ratio 455 nm / 500 nm, excited at 405 nm and 488 nm, respectively), and (**C**, **D**) EGF-CoGFP-mCRISPRed (emission ratio 500 nm / 590 nm, both excited at 458 nm). (**A**, **C**) Three lines were drawn through a region of interest in each pH map. (**B**, **D**) pH profiles P1-P3 (black, red and blue) along these lines were plotted to estimate the pH gradient among the intracellular compartments. The difference between minimal and maximal pH resulted in ΔpH values: (**B**) 1.07 (P1, black), 1.68 (P2, red), 0.97 and 0.77 (P3, blue line over two vesicles); (**D**) 0.82 (P1, black), 0.83 (P2, red), 0.48 (P3, blue).

Figure S21. Determination of transfection efficiency using flow cytometry.

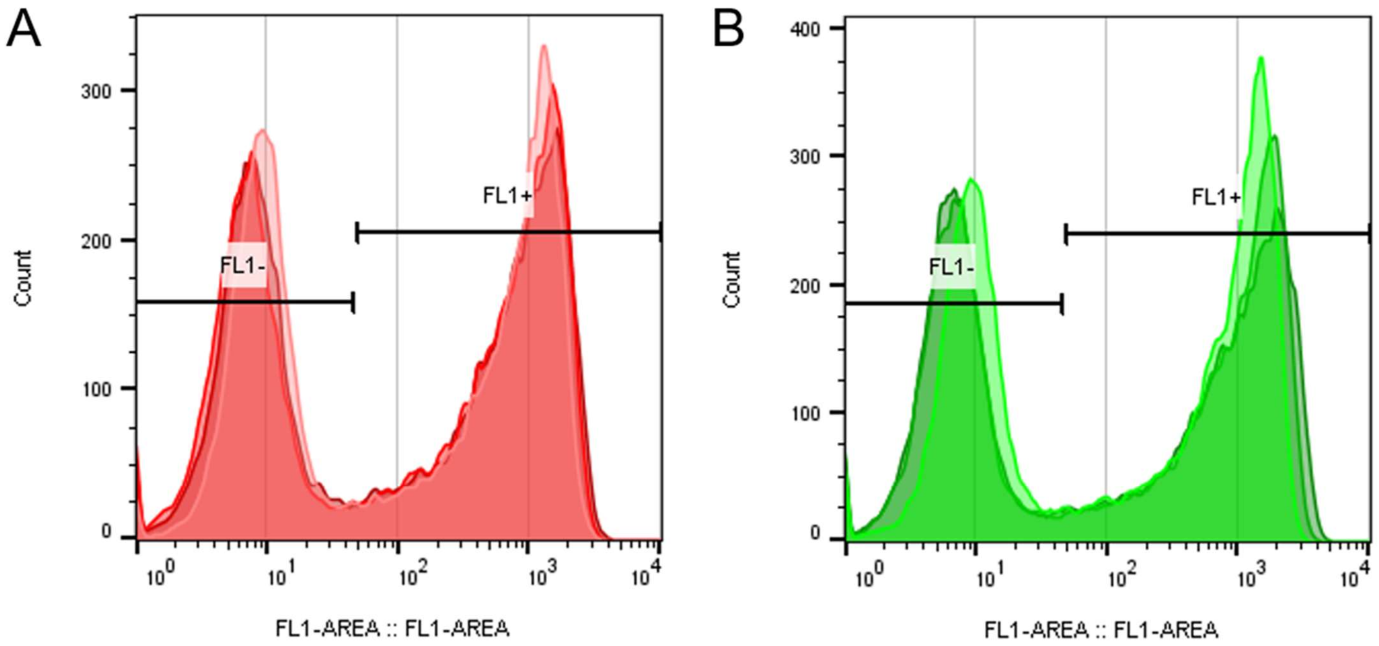

	Sample	Concentration	Freq. FL1-	Freq. FL1+	Mean FL1+
	EGF-CoGFP-mCRISPRed	100 nM	43.5%	56.2%	936
	EGF-CoGFP-mCRISPRed	200 nM	42.3%	57.5%	991
	EGF-CoGFP-mCRISPRed	500 nM	42.7%	57.0%	1025

	Sample	Concentration	Freq. FL1-	Freq. FL1+	Mean FL1+
	EGF-CoGFP-mTagBFP2	100 nM	42.3%	57.4%	1049
	EGF-CoGFP-mTagBFP2	200 nM	43.5%	56.3%	1196
	EGF-CoGFP-mTagBFP2	500 nM	44.0%	55.8%	1283

Figure S21. Determination of transfection efficiency using flow cytometry. HEK 293-F suspension cells were transfected with pcDNA6A-EGFR ECD (1-644) from Mien-Chie Hung (Addgene plasmid #42666) to express the EGFR ectodomain I-IV on the cell surface [23]. Transfection efficiency was measured 48 h post transfection. 500 µL of suspension cell culture with 3.7×10^6 cells/mL was incubated for 5 min at 22°C with 100, 200, or 500 nM of (**A**) EGF-CoGFP-mCRISPRed or (**B**) EGF-CoGFP-mTagBFP2, washed thrice with PBS (pH 7.4, 4°C) and analyzed by flow cytometry. Histograms of fluorescence intensities revealed about 56 % transfected cells (FL1+). Green fluorescence intensity (FL1: ex. 488 nm, em. 525/30 nm) for transfected cells was roughly 100-fold than observed for untransfected cells (FL1-). Concentrations ranging from 100-500 nM showed neglectable differences of mean fluorescence intensities.

2. Amino Acid Sequences

2.1. CoGFP_V0-His6

MASIPENSGLTEEMPAQMNLEGVVNGHAFSMEGIGGGNILTGIQKLDIRVIEGDP LPFSFDILSVAFQYGNRTYTSYPAKIPDYFVQSFPEGFTFERTLSFEDGAIVKVESDISIED GKFVGKIKYNGEGFPEDGPVMKKEVTKLEPSSESMYVSDGTLVGEVVLSYKTQSTHYT CHMKTIYRSKKPVELPKFHYVHHRLEKKIVEEGYYYEQHETAIAKPKLAAALEH-HHHHH

2.2. EGF-CoGFP-His6

MASMNSDSECPLSHDGYCLHDGVCMYIEALDKYACNCVVGYIGERCQYRDLK WWELRGGGSGGGSSIPENSGLTEEMPAQMNLEGVVNGHAFSMEGIGGGNILTGIQK LDIRVIEGDPLPFSFDILSVAFQYGNRTYTSYPAKIPDYFVQSFPEGFTFERTLSFEDGAIV KVESDISIEDGKFVGKIKYNGEGFPEDGPVMKKEVTKLEPSSESMYVSDGTLVGEVVLS YKTQSTHYTCHMKTIYRSKKPVENLPKFHYVHHRLEKKIVEEGYYYEQHETAIAKP-HHHHHH

2.3. EGF-CoGFP-mTagBFP2

MNSDSECPLSHDGYCLHDGVCMYIEALDKYACNCVVGYIGERCQYRDLKWWE LRGGGSGGGSSIPENSGLTEEMPAQMNLEGVVNGHAFSMEGIGGGNILTGIQKLDIRV IEGDPLPFSFDILSVAFQYGNRTYTSYPAKIPDYFVQSFPEGFTFERTLSFEDGAIVKVES DISIEDGKFVGKIKYNGEGFPEDGPVMKKEVTKLEPSSESMYVSDGTLVGEVVLSYKTQ STHYTCHMKTIYRSKKPVENLPKFHYVHHRLEKKIVEEGYYYEQHETAIAKPGSLRSVS KGEELIKENMHMKLYMEGTVDNHHFKCTSEGEGKPYEGTQTMRIKVVEGGPLPFAF DILATSFLYGSKTFINHTQGIPDFFKQSFPEGFTWERVTTYEDGGVLTATQDTSLQDGC LIYNVKIRGVNFTSNGPVMQKKTLGWEAFTETLYPADGGLEGRNDMALKLVGGSHLI ANAKTTYRSKKPAKNLKMPGVYYVDYRLERIKEANNETYVEQHEVAVARYCDLPSK LGHKLNHHHHHH

2.4. EGF-CoGFP-mCRISPRed

MNSDSECPLSHDGYCLHDGVCMYIEALDKYACNCVVGYIGERCQYRDLKWWE LRGGGSGGGSSIPENSGLTEEMPAQMNLEGVVNGHAFSMEGIGGGNILTGIQKLDIRV IEGDPLPFSFDILSVAFQYGNRTYTSYPAKIPDYFVQSFPEGFTFERTLSFEDGAIVKVES DISIEDGKFVGKIKYNGEGFPEDGPVMKKEVTKLEPSSESMYVSDGTLVGEVVLSYKTQ STHYTCHMKTIYRSKKPVENLPKFHYVHHRLEKKIVEEGYYYEQHETAIAKPGSLRSVS KGEELIKENMRMKVVMEGSVNGHQFKCTGEGEGRPYEGVQTMRIKVIEGGPLPFAFD ILATSFMYGSRTFIKYPADIPDFFKQSFPEGFTWERVTRYEDGGVVTVTQDTSLEDGELV YNVKVRGVNFPSNGPVMQKKTKGWEADTEMMYPADGGLRGYLDRALKVDGGGHL HCNFVTTYRSKKTVGDIKMPGVHAVDHRLERIEESDNETYVVQREVAVAKYSNLGG GMDELYKHHHHHH

References

1. Feiner, R.C.; Pennè, I.; Müller, B.; Müller, K.M. EGF-mCherry Fusion Protein Expressed in E. coli Shows Product Heterogeneity but a High Biological Activity. *Biochemistry* **2019**, *58*, 1043–1047. https://doi.org/10.1021/acs.biochem.9b00021.
2. Gross, L.A.; Baird, G.S.; Hoffman, R.C.; Baldridge, K.K.; Tsien, R.Y. The structure of the chromophore within DsRed, a red fluorescent protein from coral. *Proc. Natl. Acad. Sci. U. S. A.* **2000**, *97*, 11990–11995. https://doi.org/10.1073/pnas.97.22.11990.
3. Merzlyak, E.M.; Goedhart, J.; Shcherbo, D.; Bulina, M.E.; Shcheglov, A.S.; Fradkov, A.F.; Gaintzeva, A.; Lukyanov, K.A.; Lukyanov, S.; Gadella, T.W.J.; et al. Bright monomeric red fluorescent protein with an extended fluorescence lifetime. *Nat. Methods* **2007**, *4*, 555–557. https://doi.org/10.1038/nmeth1062.
4. Wiedenmann, J.; Schenk, A.; Rocker, C.; Girod, A.; Spindler, K.-D.; Nienhaus, G.U. A far-red fluorescent protein with fast maturation and reduced oligomerization tendency from Entacmaea quadricolor (Anthozoa, Actinaria). *Proc. Natl. Acad. Sci.* **2002**, *99*, 11646–11651. https://doi.org/10.1073/pnas.182157199.
5. Erdogan, M.; Fabritius, A.; Basquin, J.; Griesbeck, O. Targeted In Situ Protein Diversification and Intra-organelle Validation in Mammalian Cells. *Cell Chem. Biol.* **2020**, *27*, 610–621.e5. https://doi.org/10.1016/j.chembiol.2020.02.004.
6. Subach, O.M.; Cranfill, P.J.; Davidson, M.W.; Verkhusha, V. V. An Enhanced Monomeric Blue Fluorescent Protein with the High Chemical Stability of the Chromophore. *PLoS One* **2011**, *6*, e28674. https://doi.org/10.1371/journal.pone.0028674.
7. Suzuki, T.; Arai, S.; Takeuchi, M.; Sakurai, C.; Ebana, H.; Higashi, T.; Hashimoto, H.; Hatsuzawa, K.; Wada, I. Development of Cysteine-Free Fluorescent Proteins for the Oxidative Environment. *PLoS One* **2012**, *7*, e37551. https://doi.org/10.1371/journal.pone.0037551.
8. Chu, J.; Oh, Y.; Sens, A.; Ataie, N.; Dana, H.; Macklin, J.J.; Laviv, T.; Welf, E.S.; Dean, K.M.; Zhang, F.; et al. A bright cyan-excitable orange fluorescent protein facilitates dual-emission microscopy and enhances bioluminescence imaging in vivo. *Nat. Biotechnol.* **2016**, *34*, 760–767. https://doi.org/10.1038/nbt.3550.

9. Fabritius, A.; Ng, D.; Kist, A.M.; Erdogan, M.; Portugues, R.; Griesbeck, O. Imaging-Based Screening Platform Assists Protein Engineering. *Cell Chem. Biol.* **2018**, *25*, 1554–1561.e8. https://doi.org/10.1016/j.chembiol.2018.08.008.
10. Wannier, T.M.; Gillespie, S.K.; Hutchins, N.; McIsaac, R.S.; Wu, S.-Y.; Shen, Y.; Campbell, R.E.; Brown, K.S.; Mayo, S.L. Monomerization of far-red fluorescent proteins. *Proc. Natl. Acad. Sci.* **2018**, *115*, E11294–E11301. https://doi.org/10.1073/pnas.1807449115.
11. Subach, F. V.; Patterson, G.H.; Renz, M.; Lippincott-Schwartz, J.; Verkhusha, V. V. Bright Monomeric Photoactivatable Red Fluorescent Protein for Two-Color Super-Resolution sptPALM of Live Cells. *J. Am. Chem. Soc.* **2010**, *132*, 6481–6491. https://doi.org/10.1021/ja100906g.
12. Pennacchietti, F.; Serebrovskaya, E.O.; Faro, A.R.; Shemyakina, I.I.; Bozhanova, N.G.; Kotlobay, A.A.; Gurskaya, N.G.; Bodén, A.; Dreier, J.; Chudakov, D.M.; et al. Fast reversibly photoswitching red fluorescent proteins for live-cell RESOLFT nanoscopy. *Nat. Methods* **2018**, *15*, 601–604. https://doi.org/10.1038/s41592–018-0052–9.
13. Lambert, T.J. FPbase: a community-editable fluorescent protein database. *Nat. Methods* **2019**, *16*, 277–278. https://doi.org/10.1038/s41592–019-0352–8.
14. Ogoh, K.; Kinebuchi, T.; Murai, M.; Takahashi, T.; Ohmiya, Y.; Suzuki, H. Dual-color-emitting green fluorescent protein from the sea cactus Cavernularia obesa and its use as a pH indicator for fluorescence microscopy. *Luminescence* **2013**, *28*, 582–591. https://doi.org/10.1002/bio.2497.
15. Davidson, N.E.; Gelmann, E.P.; Lippman, M.E.; Dickson, R.B. Epidermal Growth Factor Receptor Gene Expression in Estrogen Receptor-Positive and Negative Human Breast Cancer Cell Lines. *Mol. Endocrinol.* **1987**, *1*, 216–223. https://doi.org/10.1210/mend-1–3-216.
16. Shimozono, S.; Hosoi, H.; Mizuno, H.; Fukano, T.; Tahara, T.; Miyawaki, A. Concatenation of Cyan and Yellow Fluorescent Proteins for Efficient Resonance Energy Transfer. *Biochemistry* **2006**, *45*, 6267–6271. https://doi.org/10.1021/bi060093i.
17. Wiedenmann, J.; Vallone, B.; Renzi, F.; Nienhaus, K.; Ivanchenko, S.; Röcker, C.; Nienhaus, G.U. Red fluorescent protein eqFP611 and its genetically engineered dimeric variants. *J. Biomed. Opt.* **2005**, *10*, 014003. https://doi.org/10.1117/1.1854680.
18. Kredel, S.; Oswald, F.; Nienhaus, K.; Deuschle, K.; Röcker, C.; Wolff, M.; Heilker, R.; Nienhaus, G.U.; Wiedenmann, J. mRuby, a Bright Monomeric Red Fluorescent Protein for Labeling of Subcellular Structures. *PLoS One* **2009**, *4*, e4391. https://doi.org/10.1371/journal.pone.0004391.
19. Lam, A.J.; St-Pierre, F.; Gong, Y.; Marshall, J.D.; Cranfill, P.J.; Baird, M.A.; McKeown, M.R.; Wiedenmann, J.; Davidson, M.W.; Schnitzer, M.J.; et al. Improving FRET dynamic range with bright green and red fluorescent proteins. *Nat. Methods* **2012**, *9*, 1005–1012. https://doi.org/10.1038/nmeth.2171.
20. Balleza, E.; Kim, J.M.; Cluzel, P. Systematic characterization of maturation time of fluorescent proteins in living cells. *Nat. Methods* **2018**, *15*, 47–51. https://doi.org/10.1038/nmeth.4509.
21. Subach, O.M.; Vlaskina, A. V.; Agapova, Y.K.; Dorovatovskii, P. V.; Nikolaeva, A.Y.; Ivashkina, O.I.; Popov, V.O.; Piatkevich, K.D.; Khrenova, M.G.; Smirnova, T.A.; et al. LSSmScarlet, dCyRFP2s, dCyOFP2s and CRISPRed2s, Genetically Encoded Red Fluorescent Proteins with a Large Stokes Shift. *Int. J. Mol. Sci.* **2021**, *22*, 12887. https://doi.org/10.3390/ijms222312887.
22. Schindelin, J.; Rueden, C.T.; Hiner, M.C.; Eliceiri, K.W. The ImageJ ecosystem: An open platform for biomedical image analysis. *Mol. Reprod. Dev.* **2017**, *82*, 518–529. https://doi.org/10.1002/mrd.22489.
23. Hsu, S.-C.; Hung, M.-C. Characterization of a novel tripartite nuclear localization sequence in the EGFR family. *J. Biol. Chem.* **2007**, *282*, 10432–10440. https://doi.org/10.1074/jbc.M610014200.
